# SARS-CoV-2 Mutant Spectra at Different Depth Levels Reveal an Overwhelming Abundance of Low Frequency Mutations

**DOI:** 10.3390/pathogens11060662

**Published:** 2022-06-08

**Authors:** Brenda Martínez-González, María Eugenia Soria, Lucía Vázquez-Sirvent, Cristina Ferrer-Orta, Rebeca Lobo-Vega, Pablo Mínguez, Lorena de la Fuente, Carlos Llorens, Beatriz Soriano, Ricardo Ramos-Ruíz, Marta Cortón, Rosario López-Rodríguez, Carlos García-Crespo, Pilar Somovilla, Antoni Durán-Pastor, Isabel Gallego, Ana Isabel de Ávila, Soledad Delgado, Federico Morán, Cecilio López-Galíndez, Jordi Gómez, Luis Enjuanes, Llanos Salar-Vidal, Mario Esteban-Muñoz, Jaime Esteban, Ricardo Fernández-Roblas, Ignacio Gadea, Carmen Ayuso, Javier Ruíz-Hornillos, Nuria Verdaguer, Esteban Domingo, Celia Perales

**Affiliations:** 1 Department of Clinical Microbiology, Instituto de Investigación Sanitaria-Fundación Jiménez Díaz University Hospital, Universidad Autónoma de Madrid (IIS-FJD, UAM), Av. Reyes Católicos 2, 28040 Madrid, Spain; brenda.martinez@cnb.csic.es (B.M.-G.); mesoria@cbm.csic.es (M.E.S.); luciavazsir@gmail.com (L.V.-S.); rebeccalobo10@gmail.com (R.L.-V.); llanos.salar@quironsalud.es (L.S.-V.); mario.esteban@quironsalud.es (M.E.-M.); jesteban@fjd.es (J.E.); rfernandez@fjd.es (R.F.-R.); igadea@fjd.es (I.G.); 2 Department of Molecular and Cell Biology, Centro Nacional de Biotecnología (CNB-CSIC), Consejo Superior de Investigaciones Científicas (CSIC), Campus de Cantoblanco, 28049 Madrid, Spain; l.enjuanes@cnb.csic.es; 3 Centro de Biología Molecular “Severo Ochoa” (CSIC-UAM), Consejo Superior de Investigaciones Científicas (CSIC), Campus de Cantoblanco, 28049 Madrid, Spain; carlos.garciac@cbm.csic.es (C.G.-C.); pilarsomovillacrespo@gmail.com (P.S.); tonidp09@gmail.com (A.D.-P.); igallego@cbm.csic.es (I.G.); aideavila@cbm.csic.es (A.I.d.Á.); 4 Centro de Investigación Biomédica en Red de Enfermedades Hepáticas y Digestivas (CIBERehd), Instituto de Salud Carlos III, 28029 Madrid, Spain; jgomez@ipb.csic.es; 5 Structural Biology Department, Institut de Biología Molecular de Barcelona CSIC, 08028 Barcelona, Spain; cfocri@ibmb.csic.es (C.F.-O.); nvmcri@ibmb.csic.es (N.V.); 6 Department of Genetics & Genomics, Instituto de Investigación Sanitaria-Fundación Jiménez Díaz University Hospital, Universidad Autónoma de Madrid (IIS-FJD, UAM), Av. Reyes Católicos 2, 28040 Madrid, Spain; pablo.minguez@quironsalud.es (P.M.); ldelafuente.lorena@gmail.com (L.d.l.F.); mcorton@quironsalud.es (M.C.); rosario.lopezr@quironsalud.es (R.L.-R.); cayuso@fjd.es (C.A.); 7 Centre for Biomedical Network Research on Rare Diseases (CIBERER), Instituto de Salud Carlos III, 28029 Madrid, Spain; 8 Bioinformatics Unit, Instituto de Investigación Sanitaria-Fundación Jiménez Díaz University Hospital, Universidad Autónoma de Madrid (IIS-FJD, UAM), 28040 Madrid, Spain; 9 Biotechvana, “Scientific Park”, Universidad de Valencia, 46980 Valencia, Spain; carlos.llorens@biotechvana.com (C.L.); beatriz.soriano@biotechvana.com (B.S.); 10 Unidad de Genómica, “Scientific Park of Madrid”, Campus de Cantoblanco, 28049 Madrid, Spain; ricardo.ramos@fpcm.es; 11 Departamento de Biología Molecular, Universidad Autónoma de Madrid, Campus de Cantoblanco, 28049 Madrid, Spain; 12 Departamento de Sistemas Informáticos, Escuela Técnica Superior de Ingeniería de Sistemas Informáticos (ETSISI), Universidad Politécnica de Madrid, 28031 Madrid, Spain; mariasoledad.delgado@upm.es; 13 Departamento de Bioquímica y Biología Molecular, Universidad Complutense de Madrid, 28005 Madrid, Spain; fmoran@ucm.es; 14 Unidad de Virología Molecular, Laboratorio de Referencia e Investigación en Retrovirus, Centro Nacional de Microbiología, Instituto de Salud Carlos III, Majadahonda, 28222 Madrid, Spain; ceciliolopezgalindez1462@gmail.com; 15 Instituto de Parasitología y Biomedicina ‘López-Neyra’ (CSIC), Parque Tecnológico Ciencias de la Salud, Armilla, 18016 Granada, Spain; 16 Allergy Unit, Hospital Infanta Elena, Valdemoro, 28342 Madrid, Spain; javier.ruiz@quironsalud.es; 17 Instituto de Investigación Sanitaria-Fundación Jiménez Díaz University Hospital, Universidad Autónoma de Madrid (IIS-FJD, UAM), Av. Reyes Católicos 2, 28040 Madrid, Spain; 18 Faculty of Medicine, Universidad Francisco de Vitoria, 28223 Madrid, Spain

**Keywords:** RNA virus, COVID-19, ultra-deep sequencing, viral quasispecies, mutation, deletion, nsp12 (polymerase), spike

## Abstract

Populations of RNA viruses are composed of complex and dynamic mixtures of variant genomes that are termed mutant spectra or mutant clouds. This applies also to SARS-CoV-2, and mutations that are detected at low frequency in an infected individual can be dominant (represented in the consensus sequence) in subsequent variants of interest or variants of concern. Here we briefly review the main conclusions of our work on mutant spectrum characterization of hepatitis C virus (HCV) and SARS-CoV-2 at the nucleotide and amino acid levels and address the following two new questions derived from previous results: (i) how is the SARS-CoV-2 mutant and deletion spectrum composition in diagnostic samples, when examined at progressively lower cut-off mutant frequency values in ultra-deep sequencing; (ii) how the frequency distribution of minority amino acid substitutions in SARS-CoV-2 compares with that of HCV sampled also from infected patients. The main conclusions are the following: (i) the number of different mutations found at low frequency in SARS-CoV-2 mutant spectra increases dramatically (50- to 100-fold) as the cut-off frequency for mutation detection is lowered from 0.5% to 0.1%, and (ii) that, contrary to HCV, SARS-CoV-2 mutant spectra exhibit a deficit of intermediate frequency amino acid substitutions. The possible origin and implications of mutant spectrum differences among RNA viruses are discussed.

## 1. Introduction

High mutation rates of RNA viruses have the following two major consequences: (i) that viruses are composed of (and replicate as) complex mutant distributions, also termed mutant spectra or mutant clouds, and (ii) that interactions of complementation, cooperation, or suppression among components of a mutant spectrum, exerted mainly through their expression products, can influence the behavior of the ensemble, and give rise to emergent phenotypes [1,2,3,4,5]. Suppression of infectious genomes by a mutagenized mutant spectrum is one of the ingredients that contribute to virus extinction in the process of lethal mutagenesis, presently used as an antiviral strategy [6,7,8,9,10,11,12]. Modifications of mutant spectrum composition and the intra-mutant spectrum interactions that may modulate the behavior of the entire population are referred to as quasispecies dynamics. The historical origins of the quasispecies concept and the connections between theory and experimental observations have been recently reviewed [13].

Replication as complex mutant distributions is a driver of virus diversification and adaptability—sometimes reflected in disease progression—through exploration of sequence space and supply of phenotypic variants, which are the substrate on which selection acts. The minority genomes present in a mutant spectrum can become medically and epidemiologically relevant as a result of selection or random drift, the latter prompted by bottleneck events of different intensities (as defined by the number of particles involved) [14,15,16]. Population heterogeneity extends to RNA viruses (and some DNA viruses) replicating in their natural habitats, as well as in cell culture, where designed experiments have permitted quantification of relevant parameters (mutation rates and frequencies, population size effects on viral fitness, etc.) (as representative articles and reviews, see [17,18,19,20,21,22,23,24,25,26,27,28,29,30,31]).

Coronaviruses and SARS-CoV-2 are not an exception to viral genome replication in the form of dynamic and compartmentalized (distinct in different locations of the same infected individual) mutant spectra [32,33,34,35,36,37,38,39,40,41,42,43,44,45,46]. Mutations that are present as a minority (low frequency) in an infected individual can be represented in the consensus sequence of epidemiologically distant isolates [42,47]. Moreover, the mutant spectra of isolates from vaccination-breakthrough COVID-19 cases contained previously undescribed mutations that then rose to dominance in isolates from later epidemic waves [46]. Therefore, the complexity and composition of SARS-CoV-2 mutant spectra can inform the genome composition of the virus at the population level in a realistic way and of the repertoire of tolerated mutations, the latter as data input to try to anticipate possible evolutionary trajectories of the virus [25,48]. Mutant spectrum analyses may also identify genomic regions displaying stricter conservation than concluded from an examination of consensus sequences or current data bank repositories; this information can be useful to design universal vaccines and pan-genotypic antiviral agents [49].

We have recently characterized the SARS-CoV-2 mutant spectra present in diagnostic samples from COVID-19 patients and vaccine-breakthrough infections in terms of complexity, mutation and deletion frequency, and the association of these parameters with infection severity [46,47]. In the course of these quantifications, we noted significant differences between SARS-CoV-2 and other RNA viruses—that we have studied over the last 20 years—in the frequency distribution of minority mutations. This prompted us to re-examine SARS-CoV-2 mutant spectra with a lower cut-off frequency limit and also to quantify differences with mutant spectra of other RNA viruses. In particular, the use of the same deep sequencing methodology and bioinformatics pipelines allowed a direct comparison of some proteins of hepatitis C virus (HCV) and SARS-CoV-2, including their RNA-dependent RNA polymerase. In the present report, we first review the main conclusions of our previous work on the quasispecies dynamics of HCV and mutant spectrum composition of SARS-CoV-2 diagnostic samples and then address the following two questions: (i) the numbers and types of minority mutations and deletions that can be detected in SARS-CoV-2 mutant spectra upon decreasing the limit of detection of point mutations and deletions, and (ii) quantification of the differences in mutant spectrum composition between SARS-CoV-2 and HCV regarding the repertoire of amino acid substitutions in virus from infected patients. The results suggest (i) the presence of an impressively large repository of low-frequency variants in diagnostic samples of SARS-CoV-2 and (ii) that the cloud configuration in terms of mutant frequency distribution differs between SARS-CoV-2 and HCV.

## 2. Materials and Methods

### 2.1. Origin of HCV from Chronically Infected Patients, and of HCV Populations Adapted to Human Hepatoma Cells in Culture

A standardized method to identify antiviral resistance-associated substitutions in HCV proteins was developed by Quer, Perales, and colleagues and applied to 220 subtyped samples from a cohort of patients belonging to 39 Spanish hospitals [50,51,52,53,54]. The procedures for subtyping, oligonucleotide primer design, viral RNA amplification, ultra-deep sequencing, and the bioinformatics pipelines used for mutant spectrum analysis at the amino acid level have been described [52,53,54,55].

Studies on HCV population dynamics in cell culture were carried out with clonal population HCV p0, which was derived by transcription from plasmid Jc1FLAG2(p7-nsGluc2A [56], as previously described [57]. This initial virus was subjected to 210 serial passages in human hepatoma Huh-7.5 cells. The initial, final, and intermediate passage populations were analyzed by deep sequencing, using bioinformatics pipelines adapted from those employed for the analysis of HCV from infected patients. The bioinformatics and virological procedures (infection conditions, multiplicity of infection, duration of infection, extraction of intracellular and extracellular RNA, amplification of viral RNA) have been detailed in the relevant publications [49,57,58,59,60,61].

### 2.2. COVID-19 Patient Cohort, Stratification, and Amplification of SARS-CoV-2 RNA from Diagnostic Samples

Since the procedures for SARS-CoV-2 studies have been recently implemented in our laboratory, here we describe them with greater detail than for the studies with HCV (Section 2.1). The mutant spectrum analysis of SARS-CoV-2 was performed on diagnostic, nasopharyngeal samples of 30 patients admitted to the Fundación Jiménez Díaz Hospital (FJD, Madrid, Spain) from 3 April to 29 April 2020 during the first COVID-19 outbreak in Spain. They were confirmed as positive for SARS-CoV-2 by a specific real-time RT-PCR analysis, as previously described [62]. They were classified according to the severity of their associated COVID-19 diagnosed in each infected patient; the criteria for disease severity were the following: mild (no need of hospitalization; *n* = 10); moderate (hospitalization without need of intensive care unit; *n* = 10); severe (hospitalization with requirement of intensive care unit, ending in exitus in all cases; *n* = 10). Co-morbidities that might influence disease manifestations were equally distributed among the three severity groups, as previously detailed for each patient [47,62].

RNA was extracted from 140 µL of the nasopharyngeal swab sample using the QIAamp viral RNA Mini Kit (250) from Qiagen, following the manufacturer’s instructions. Two genomic regions of SARS-CoV-2 were amplified by RT-PCR for deep sequencing analysis as follows: Nucleotides from 14,511 to 16,075 of the nsp12 (polymerase)-coding region, which correspond to amino acids from 366 to 871 of nsp12 (polymerase), and nucleotides from 22,853 to 23,666 of the spike protein (S)-coding region, which correspond to amino acids from 438 to 694 of protein S (nucleotide and amino acid residue numbering are according to reference sequence NC_045512.2). The primers used for SARS-CoV-2 RNA amplification have been previously described [47] and are listed in Supplementary Material Table S1. The amplifications were carried out using the Transcriptor One Step RT-PCR kit (Roche Applied Science, Penzberg, Germany), as follows: 5 µL of the RNA preparation (12,5% of the total volume) were mixed with 10 µL of 5× buffer, and 2 µL of a solution that contained the forward oligonucleotide primer, 2 µL of a solution that contained the reverse primer (50 ng/µL each), and 1 µL of polymerase (Transcriptor reverse transcriptase and Taq). The reverse transcription step was performed at 50 °C for 30 min, followed by an initial denaturing step at 94 °C for 7 min, and 35 cycles of a denaturing step at 94 °C for 10 s, an annealing step at 46–48 °C for 30 s, an extension step at 68 °C for 40 s, and then a final extension at 68 °C for 7 min. For samples with a Ct > 26, the number of cycles was increased to 45. Amplification controls in the absence of viral RNA were run in parallel to ensure the absence of contaminating RNA templates. The amplification products were analyzed by 2% agarose gel electrophoresis, using Gene Ruler 1 Kb Plus DNA ladder (Thermo Scientific, Waltham, MA, USA) as molar mass standard; they were purified (QIAquick Gel Extraction Kit, Qiagen, Hilden, Germany), quantified (Qubit dsDNA Assay kit, Thermofisher Scientific), and tested for quality (TapeStation System, Agilent Technologies, CA, USA) prior to sequencing using the Illumina MiSeq platform. Dilutions of 1:10, 1:100, and 1:1000 of some initial RNA preparations and subsequent amplification by RT-PCR were carried out and produced a visible DNA band. Ultra-deep sequencing analysis was performed with the amplification products of the undiluted template to avoid redundant copying of the same template molecules; these procedures for deep sequencing sample preparation have been previously described [46,50,51,52,53,54].

### 2.3. Ultra-Deep Sequencing of SARS-CoV-2

**Figure 1:**
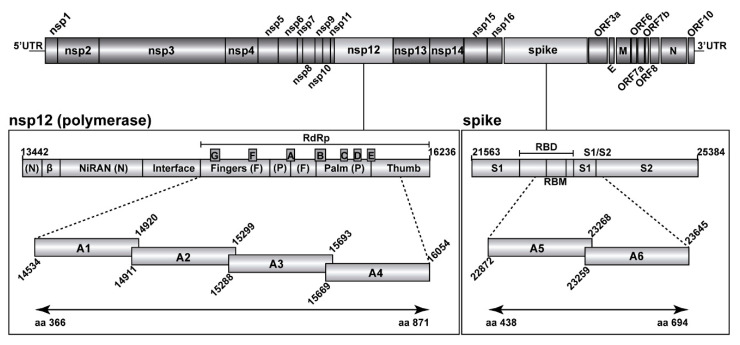
Representation of the SARS-CoV-2 genome, encoded proteins, and amplicons analyzed by UDS. In the two boxes below the scheme of the genome, the two genomic regions under study have been expanded, with genome residue numbers according to reference genome NCBI accession number NC_045512.2. The position of relevant protein domains is indicated. Left box: polymerase A to G motifs in the RdRp, and other domains of nsp12. Right box: spike (S) receptor binding motif (RBM) within the receptor binding domain (RBD), and the S1/S2 cleavage site. The amplicons analyzed in the present study are depicted as horizontal boxes [A1 to A4 for the nsp12 (polymerase) region, and A5, A6 for the S region]. Residue numbers delimiting each of the amplicons are shown, and the amino acid residues analyzed in the two proteins are indicated in the bottom lines. Materials and procedures used for amplicon preparation are detailed in Materials and Methods.

The amplification products corresponded to amplicons A1 to A4 (nsp12-coding region) and A5, A6 (S-coding region) with some overlapping sequences among neighbor amplicons (Figure 1). The concentration of the RT-PCR products was adjusted to 4 × 10^9^ molecules/µL, and DNA pools were purified using Kapa Pure Beads (Kapa Biosystems, Roche). Purified DNA was quantified using Qubit (Qubit dsDNA Assay kit, Thermofisher Scientific) as described previously [52,53,54] and adjusted to a concentration of 1.5 ng/µL. The DNA was further processed using the Kapa Hyper Prep kit (Kapa Biosystems, Roche), during which each DNA pool was indexed using the SeqCap Adapter Kit A/B (Roche) (24 Index). Each DNA pool was adjusted to 4 nM concentration to prepare the final library, which was quantified (LightCycler 480), and sequenced using the MiSeq platform with MiSeq Reagent kit v3 (2 × 300 bp mode, with the 600 cycle kit) (Illumina, CA, USA). Each run can include approximately a maximum number of amplicons of 96.

### 2.4. Bioinformatics Analyses of SARS-CoV-2 Nucleotide Sequences

Beginning with the Fastq data, we applied the bioinformatics pipeline SeekDeep [63], as previously described [46,47]. Controls to establish the basal error, the frequency of PCR-induced recombination, and the similarity of the results with different amplifications and sequencing runs using different aliquots of the same initial sample were previously performed with HCV [51,52,53,54]. Comparisons that indicate the adequacy of the adapted bioinformatics pipelines for the analysis of SARS-CoV-2 mutant spectra have been reported [47]. In particular, (88.93 ± 2.0)% of bases that we have obtained correspond to an average quality score (Q) higher than 30 (https://emea.illumina.com/systems/sequencing-platforms/miseq/specifications.html, accessed on 27 April 2022). The total number of clean reads per amplicon A1 to A6 and sample averaged 110,074 (range 89,201–129,807) with a minimum and maximum value per amplicon and sample between 39,003 and 196,471 (Table S2). This clean read coverage allowed establishing a cut-off value for mutant frequency of 0.1% and, therefore, expanding the mutant spectrum analysis of point mutations and deletions that was set at 0.5% in our previous study [47]. Validation of the 0.1% mutation frequency cut-off results relative to those previously obtained with a 0.5% cut-off [47] is further supported by the following: (i) detection of all 96 different point mutations, and all 10 different deletions found at 0.5% cut-off level also at 0.1% cut-off level, with the sole exception of mutation T15,756C in the nsp12 (polymerase)-coding region; this discrepancy has unknown reasons; (ii) a 80% agreement of the mutations (or absence of mutations) scored in the regions that overlap among each two amplicons; (iii) the maintenance of mutational bias, and of the ranking of mutation occurrence in the 3rd > 2nd > 1st codon position, and amino acid substitution acceptability scores when moving from 0.5% to 0.1% mutant frequency cut-off; (iv) 97.5% of the amino acid substitutions in the nsp12 (polymerase) and S deduced from the analysis with a 0.1% cut-off are represented in isolates deposited in the “outbreak.info” data base (as of 24 March 2022); this percentage is very similar to the one reported with a 0.5% cut-off [47]; finally, (v) a statistically significant increase in the number of amino acid substitutions with predicted functional effect according to SNAP2 (Screening for Non-Acceptable Polymorphisms 2) 1 comparing the nsp12 (polymerase)- and the spike-coding regions at 0.1% mutant frequency cut-off (Table S5); the increase would have not been statistically significant if it were due to random mistakes in the nucleotide sequence determinations. Data on points (i), (ii), (iii), (iv), and (v) are given in the Results; see also Discussion.

### 2.5. Statistics

The statistical significance of differences between the number and type of mutations in viruses from mild, moderate, and severe (exitus) COVID-19 patients, as well as the differences among types of nucleotide changes and among PAM250 (accepted point mutations 250) [64], and SNAP2 [65], were calculated by the proportion test. Differences between the distribution of frequencies in SARS-CoV-2 and HCV populations were calculated using Chi-square test with Monte Carlo correction. Statistics were inferred using software R version 4.0.2.

## 3. Results

### 3.1. A Review of Implications of HCV Population Complexity and Dynamics for Antiviral Resistance and Vaccine Efficacy

The analyses of mutant spectra of HCV isolated from patients who failed DAA-based therapies [53,54] suggest that there are antiviral resistance mechanisms alternative to the selection of standard resistance-associated substitutions (RAS). The evidence came from the identification—in basal and post-treatment failure HCV RNA samples—of a number of highly represented amino acid substitutions (termed HRSs), irrespective of the specific DAA treatment administered [53]. In these patients, treatment failure occurred in the presence of HRSs, irrespective of the presence of RAS. Similar findings have been described for other patient cohorts [54,66,67,68,69,70,71,72,73,74,75]. The mechanism of HRSs-associated antiviral resistance is unknown. One possibility is that it may relate to high viral replicative fitness, which is known to confer a general antiviral resistance phenotype. This new mechanism of antiviral resistance was documented by comparing high- and low-fitness HCV populations in cell culture [57,76,77,78]. If this mechanism operated in vivo, it would provide yet another practical implication of viral fitness, in this case as a mediator of antiviral resistance ([79]; fitness implications have been recently reviewed [80]). It is not known if antiviral resistance mechanisms different from RAS selection also operate in other RNA viruses.

A related aspect of HCV population dynamics was revealed by mutant spectrum analyses of the virus that was passaged 210 times in Huh-7.5 cells. The mutant spectrum composition was continuously modified, despite the absence of external selective pressures, suggesting that no mutation-selection equilibrium was even approached. This conclusion is based on the following two related results: the presence of mutational waves and the intra-mutant spectrum haplotype profiles. Mutational waves are defined as individual point mutations whose frequency varies and fluctuates as a function of passage number. They persisted and did not cease (rather, they increased) in late passages when the population was expected to be better adapted to the Huh-7.5 cells [58,59,60]. The haplotype distribution in a two-dimensional artificial neural network (self-organized map or SOM) [81,82] exhibited an expansion of haplotype space upon HCV passage, together with a surprising shift of the haplotype peak positions on the two-dimensional grid at passage 200 relative to their ancestors in the parental clonal population and at passage 100 [61]. Intra-mutant spectrum haplotype distributions—which are a surrogate of fitness profiles—prove highly dynamic, with no tendency to move towards a population equilibrium [61,79]. These results with HCV are in agreement with observations on fitness dynamics and plasticity of fitness landscapes obtained with other animal and plant viruses [80].

A compilation of the extended repertoire of minority mutations and amino acid substitutions in HCV from infected patients and laboratory populations established that residue conservation at the quasispecies level is less strict than conservation defined by consensus sequences or by the alignments of the Los Alamos National Laboratory data bank [49]. This implies that universal vaccines or pan-genomic antiviral agents for HCV are unlikely to be effective if their design relies on conservation deduced from sequence alignments in data banks. Vaccines and antiviral agents are directed at dynamic mutant spectra, not at a static genome as portrayed in consensus sequences or data banks. Although it is not known whether the conclusions reached with HCV will hold for other viral pathogens, the results offer yet another motivation to penetrate into the mutant spectrum composition of pathogenic viruses and investigate their biological consequences. We are currently pursuing this line of research with SARS-CoV-2, including among our objectives a comparison of its mutant spectrum profile with that of HCV.

### 3.2. Ultra-Deep Sequencing Analysis at 0.1% Cut-Off SARS-CoV-2 Mutant Spectra from Patients Progressing towards Different COVID-19 Severity

We previously described ultra-deep sequencing analyses of SARS-CoV-2 obtained from nasopharyngeal swabs using a cut-off mutant and a deletion frequency value of 0.5% [47]. Interestingly, all point mutations and deletions were found at frequencies below 30%, excluding mutations that were dominant relative to the sequence of the Wuhan-Hu-1 isolate (NCBI reference sequence NC_045512.2) that was taken as a reference [83]. We use the term dominant (also called “Divergence”) to refer to those mutations with a frequency between 90% and 100% and that, therefore, modify the consensus sequence of the isolate [47]. To penetrate deeper into the composition of the mutant spectra, and given the clean read coverage attained (Table S2), here we report the point mutation and deletion repertoire of the same SARS-CoV-2 mutant spectra using a 0.1% cut-off value, as described in Materials and Methods.

**Figure 2:**
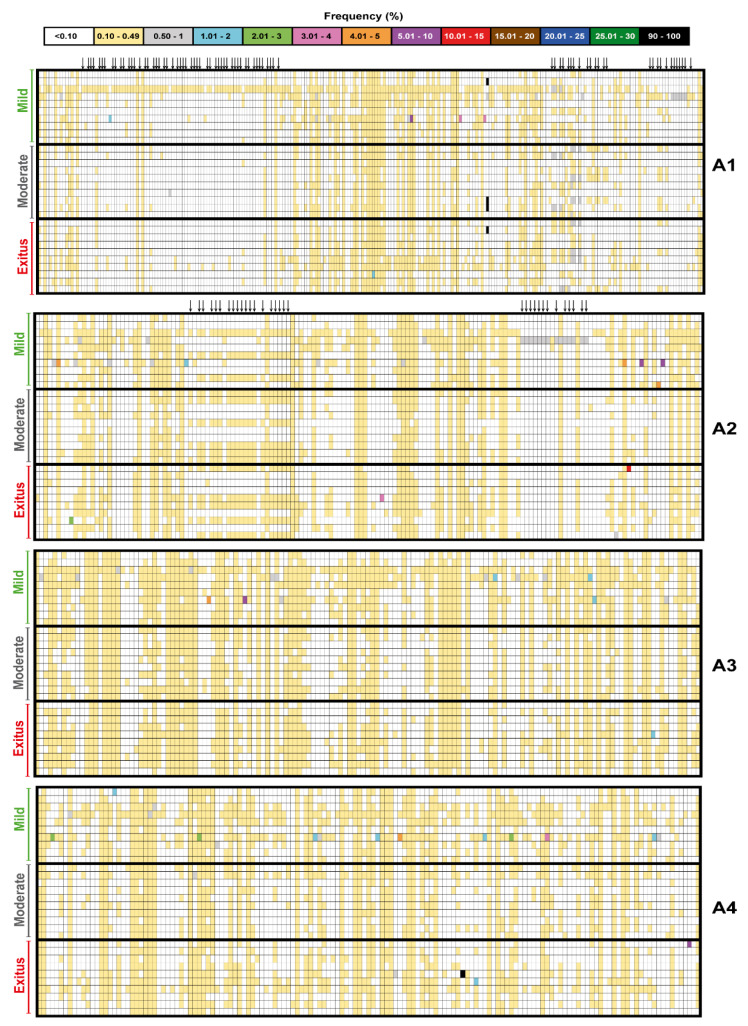
Heat map of point mutation and deletion frequencies for the nsp12 (polymerase)-coding region (genomic residues 14,534–16,054) in mutant spectra of SARS-CoV-2 from individual patients with a cut-off value of 0.1%, divided according to associated COVID-19 severity (indicated on the left of each map). Data are presented in four blocks that correspond to the four amplicons (A1 to A4); the genomic residues spanned by each amplicon are shown in Figure 1. Only positions with a mutation or those affected by a deletion (arrow symbols at the top of each block) are represented; the complete list of mutations, their position, type, deduced amino acid substitutions, their acceptability, and association with disease severity, are listed in Tables S3 and S4. The mutant frequency has been visualized with a color code displayed in the heading box. Each row corresponds to a patient whose clinical profile and identification code were previously reported [47]. Mutations and deletions have been identified relative to NCBI reference sequence NC_045512.2. Procedures are detailed in Materials and Methods.

**Figure 3:**
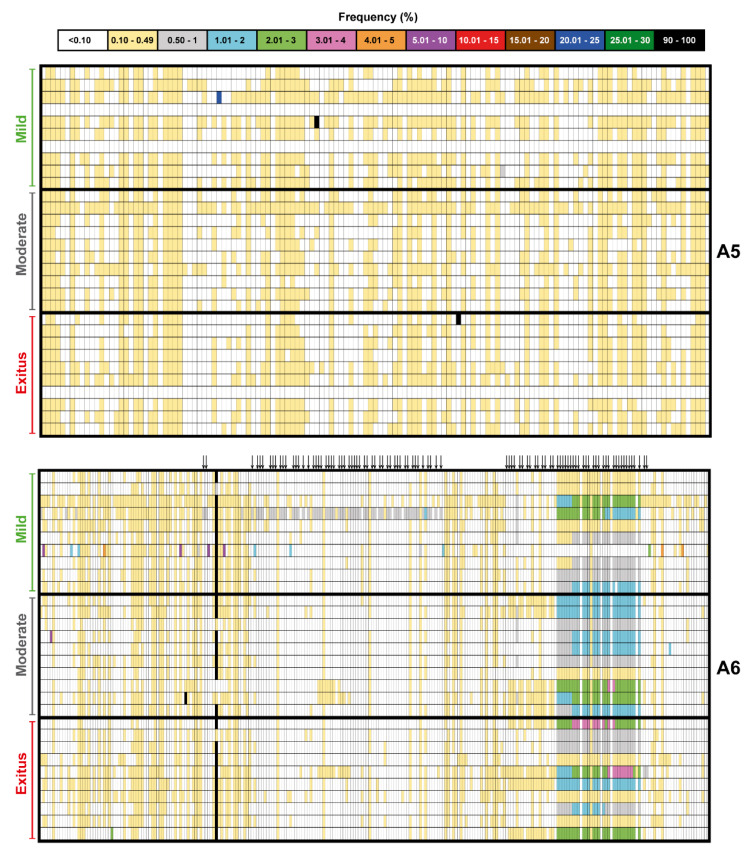
Heat map of point mutation and deletion frequencies for the spike-coding region (genomic residues 22,872–23,645) in mutant spectra of SARS-CoV-2 from individual patients with a cut-off value of 0.1%, divided according to associated COVID-19 severity (indicated on the left of each map). Data are presented in two blocks that correspond to the two amplicons (A5 to A6); the genomic residues spanned by each amplicon are shown in Figure 1. Only positions with a mutation or those affected by a deletion (arrow symbols at the top of the lower block) are represented; the complete list of mutations, their position, type, deduced amino acid substitutions, their acceptability, and association with disease severity, are listed in Tables S3 and S4. The mutant frequency has been visualized with a color code displayed in the heading box. Each row corresponds to a patient whose clinical profile and identification code were previously reported [47]. Mutations and deletions have been identified relative to NCBI reference sequence NC_045512.2. Procedures are detailed in Materials and Methods.

The mutant spectrum heterogeneity in the virus from each of the 30 patients (divided according to associated disease severity) was visualized by constructing a frequency heat map of point mutations and deletions for each amplicon of the nsp12 (polymerase) and S-coding regions (Figure 2 and Figure 3). Mutations and deletions were counted relative to the sequence of the Wuhan-Hu-1 isolate (NCBI reference sequence NC_045512.2), which was used as a reference. For each isolate, the heat map is very similar to that obtained by counting the mutations relative to the consensus sequence of the corresponding isolate. This is because the number of mutations that fall in the 90–100% frequency range—and that modify the consensus sequence of the isolate relative to the reference (black symbols in the heat maps of Figure 2 and Figure 3)—is very small. The percentage of positions with a mutation amounts to 38.5% for the nsp12 (polymerase)-coding region and 38.7% for the S-coding region.

### 3.3. A Comparison of the SARS-CoV-2 Point Mutation and Deletion Repertoire at 0.5% and 0.1% Frequency Cut-Off

**Figure 4:**
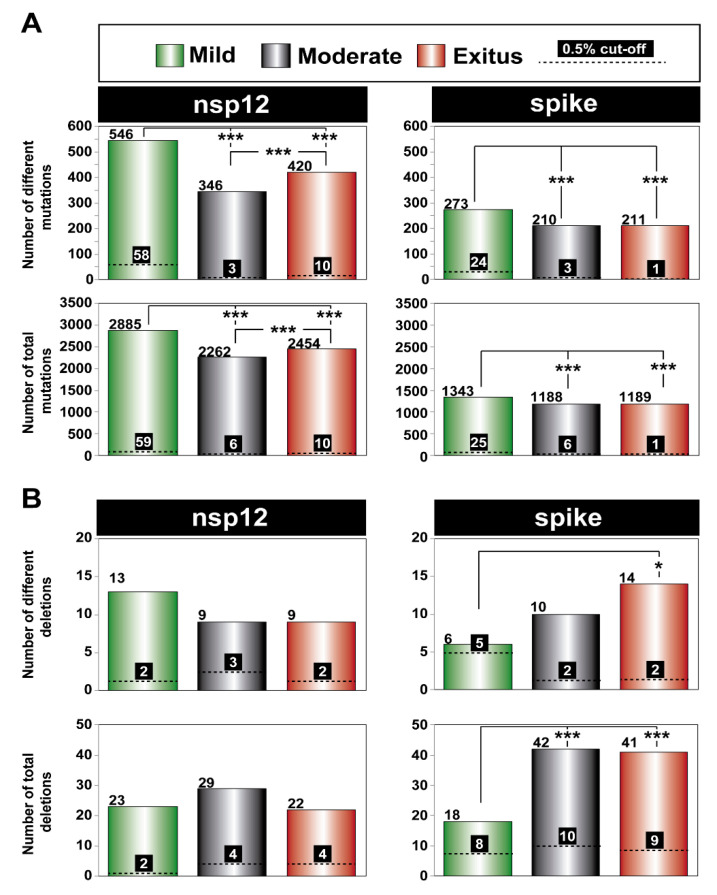
Number of genetic lesions in mutant spectra of SARS-CoV-2, distributed according to associated COVID-19 severity, determined at 0.5% and 0.1% cut-off frequency (codes in upper box). The genomic region is indicated at the top of each panel group, and the amplicons are depicted in Figure 1. (**A**) Number of different and total point mutations. The number of mutations determined with a 0.1% frequency cut-off are indicated on top of each bar, and the number previously determined with a 0.5% cut-off frequency [47] is given above the discontinuous horizontal line within each bar. (**B**) Same as (**A**) but for deletions. The complete information of mutations and deletions is listed in Tables S3 and S4. Only statistically significant differences in the number of mutations or deletions are shown (*, *p* < 0.05; ***, *p* < 0.001; proportion test).

Upon lowering the cut-off frequency value from 0.5% to 0.1%, the increase in the number of different mutations in the SARS-CoV-2 mutant spectra averaged 55-fold for the nsp12 (polymerase) and 97-fold for the S-coding region. The corresponding values for the total number of mutations were 223-fold and 480-fold, respectively (Figure 4A). The striking contrast between the different and total number of mutations scored with the 0.1% (but not with the 0.5%) cut-off is due to the increased number of different haplotypes that have been revealed by the 0.1% frequency cut-off and that harbor the same mutation. The complete list of point mutations identified in the different amplicons is listed in Table S3. The significantly higher frequency of mutations in the virus from patients displaying mild than moderate or severe disease [47] was maintained with the 0.1% cut-off frequency.

The increase in the number of different deletions in moving from the 0.5% to 0.1% cut-off was more modest, amounting to 5-fold and 4-fold for the nsp12 (polymerase) and S-coding region, respectively. The corresponding values for the total number of deletions were 8-fold and 4-fold, respectively (Figure 4B). The complete list of deletions identified in the different amplicons is listed in Table S4. In the nsp12 (polymerase)-coding region, no significant differences were observed in the number of different or total deletions among samples corresponding to the three disease categories (Figure 4B), using the 0.1% deletion frequency cut-off. Significant differences in favor of a larger number of deletions in viruses from patients that suffered moderate and severe disease than in viruses from patients who displayed mild symptoms were noted in the S-coding region (Figure 4B). This difference did not reach statistical significance when the deletion frequency cut-off was established at 0.5% [47]. Concerning the number of deletions that generated a stop codon, it was significantly higher in the S-coding region (82 out of 101 deletions) than in the nsp12 (polymerase)-coding region (21 out of 74 deletions) (*p* < 0.001; proportion test), in agreement with the conclusion of our previous study with the 0.5% deletion frequency cut-off [47]. In summary, moving from a 0.5% to a 0.1% frequency cut-off greatly increased the repertoire of minority point mutations identified in mutant spectra and also the number of deletions, albeit to a lesser extent.

### 3.4. SARS-CoV-2 Mutation and Deletion Repertoires at Progressively Lower Detection Limit

**Figure 5:**
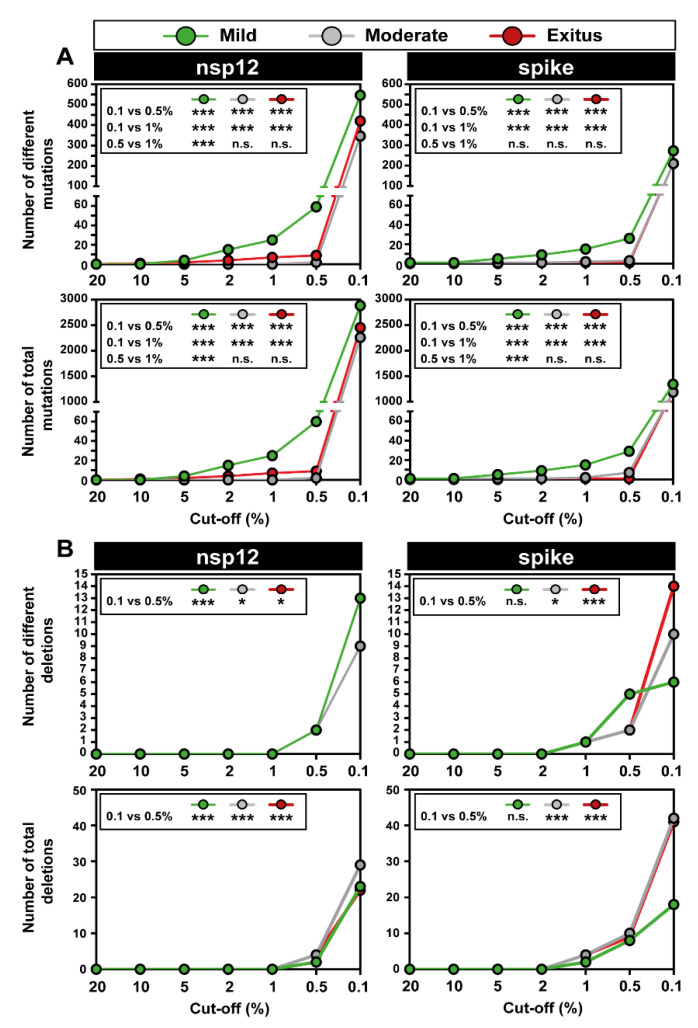
Number of point mutations and deletions detected with different frequency cut-off values in diagnostic samples of SARS-CoV-2, grouped according to associated COVID-19 severity (color code in upper box). The genomic region is indicated at the top of each panel group. Insets include the statistical significance of relevant differences. (**A**) Number of different and total point mutations, as indicated in ordinate. The complete list of point mutations detected with a 0.1% frequency cut-off is given in Table S3. (**B**) Number of different and total deletions, as indicated in ordinate. The complete list of deletions detected with a 0.1% frequency cut-off is given in Table S4, and their location in the genomic regions is depicted in Figure S1. Experimental and bioinformatics procedures are described in Materials and Methods. Statistically significant differences in the number of mutations or deletions are shown (n.s., *p* > 0.05; *, *p* < 0.05; ***, *p* < 0.001; proportion test).

The analysis of deep sequencing data was completed by comparing the number of mutations and deletions that were identified when setting the cut-off frequency value at 20%, 10%, 5%, 2%, 1%, 0.5%, and 0.1%. This provided a visualization of the dramatic overabundance of low-frequency mutations and deletions, with their statistical support (Figure 5). The results with the series of cut-off levels below 1% confirm that the mutation abundance in SARS-CoV-2 mutant spectra is consistently larger for isolates associated with mild than moderate or severe COVID-19, as previously reported [47]. The corresponding numbers of deletions [that span from 2 to 52 nucleotides in the nsp12 (polymerase)-coding region and from 2 to 51 nucleotides in the S-coding region] are displayed in Figure S1 with an indication of the number of patients in whom each deletion was found. The overwhelming abundance of low-frequency point mutations and deletions in SARS-CoV-2 mutant spectra begs for investigation of their origin and their significance as potential reservoirs of alternative phenotypes (see Discussion).

The high transition to transversion ratio (34 for a number of different mutations in the nsp12-coding region and 26 in the S-coding region) that was calculated with the 0.5% cut-off frequency [47] was accentuated when the mutations in the 0.10–0.49 frequency range entered the calculation; the new ratios were 96 and 149, respectively (Table 1). The bias is more evident when the total number of point mutations is considered (ratio of 542 for the nsp12 region and 1859 for the S region), and it applies to the isolates of the three disease categories (Table 1).

In both coding regions analyzed, the preferred types of mutation types observed with the 0.1% frequency cut-off were T → C > A → G > C → T, coincident with the preferences when the frequency cut-off was 0.5% [47], with statistically significant differences within each COVID-19 category (Figure S2). 

The dominance of non-synonymous versus synonymous mutations was maintained with the 0.1% cut-off frequency, with statistical significance in viruses from each COVID-19 category. These data further confirm the results recorded at the 0.5% cut-off frequency [47] (Table 2).

### 3.5. Acceptability of the Low Frequency Amino Acid Substitutions

We examined the acceptability of the repertoire of amino acid substitutions identified in nsp12 (polymerase) and S using the 0.1% mutation frequency cut-off. The comparison of the two proteins revealed a significantly larger proportion of substitutions with a predicted functional effect (SNAP2 algorithm > 0, according to [65]) for nsp12 (49.13% of the total) than for S (32.37% of the total). This difference did not reach statistical significance when the cut-off was set at 0.5% (Table S5). Other comparisons—such as the frequency of substitutions with low acceptability (PAM 250 < 0, according to [64]) between the two proteins, or in the same protein at 0.1% and 0.5% cut-off, or the proportion of substitutions with SNAP2 > 0 for the same protein comparing the 0.1% and 0.5% cut-off—did not reveal statistically significant differences (Table S5).

The point mutation and deletion repertoire accessed with a 0.1% frequency cut-off has shown an impressively large number of minority point mutations spanning the frequency range of 0.1–0.49% and has confirmed the absence of mutations and deletions at intermediate and high frequencies (30–90% range) [47]. Biases in the types of mutations were similar using the 0.1% or 0.5% cut-off frequency. Decreasing the cut-off to 0.1% suggested a larger repertoire of substitutions with predicted functional effects in nsp12 (polymerase) than in S. In view of these results, we sought to compare the SARS-CoV-2 mutant spectrum profile with that of HCV from chronically infected patients.

### 3.6. Comparison of HCV and SARS-CoV-2 Mutant Spectra

**Figure 6:**
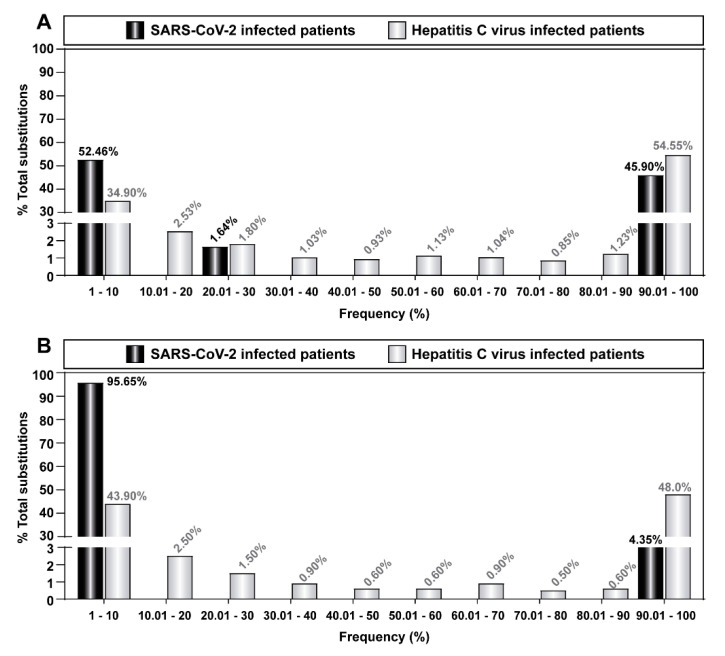
Percentage of amino acid substitutions in SARS-CoV-2 and HCV populations sampled from infected patients that fall in each frequency range, with a 1% as the low frequency limit. The virus identification code is given in the upper boxes. (**A**) Distribution of amino acid substitutions deduced from all amplicons analyzed (A1 to A6 for SARS-CoV-2 as depicted in Figure 1, and amplicons corresponding to proteins NS3, NS5A, and NS5B for HCV, as described in [51,52,53,54]); the complete list of SARS-CoV-2 amino acid substitutions is given in Table S3 and the complete list of HCV is given in Table S1 of [53]. (**B**) Same as (**A**) but for the comparison restricted to the two polymerase proteins, nsp12 for SARS-CoV-2, and NS5B for HCV. Data origin, experimental procedures, and bioinformatics pipelines are described in Materials and Methods.

We previously characterized the spectrum of amino acids in 220 several HCV serum isolates belonging to a Spanish cohort of chronically infected patients; the substitutions were counted relative to the HCV reference subtype of each isolate [51,52,53,54,84]. These studies with HCV used the bioinformatics pipelines that have now been adapted to the analysis of SARS-CoV-2 in diagnostic samples from COVID-19 patients [47]. The data are available with a 1% amino acid substitutions low limit frequency level, and they afforded an opportunity to the evaluation of whether the frequency repertoire of amino acid substitutions differed between SARS-CoV-2 and HCV. When comparing the amino acid substitution deduced from all amplicons [corresponding to proteins NS3, NS5A, and NS5B for HCV; proteins nsp12 (polymerase) and S for SARS-CoV-2], the percentage of substitutions that fell in the 10% to 90% frequency range was 10.5% for HCV and 1.6% for SARS-CoV-2 (Figure 6A). When the comparison was restricted to the polymerase [NS5B for HCV; nsp12 (polymerase) for SARS-CoV-2], the values were 8.1% for HCV and 0% for SARS-CoV-2 (Figure 6B).

The deficit of intermediate frequency substitutions in SARS-CoV-2 as compared with HCV was statistically significant [*p* = 0.025 when all proteins analyzed were considered (Figure 6A), and *p* < 0.001 when the polymerases were considered (Figure 6B); Chi-square test with Monte Carlo correction], in agreement with the results obtained at the nucleotide level (compare Figure 2, Figure 3 and Figure 6). Given the different number of isolates of the two viruses involved in the comparison, we repeated the comparison of mutant frequency distribution using the same 30 SARS-CoV-2 isolates but only a subset of either 72 (all basal HCV samples), or 39 HCV samples (the subset of basal samples that correspond to subtype G1b). In all cases, the deficit of intermediate frequency substitutions in SARS-CoV-2 was statistically significant (*p* < 0.001 to *p* = 0.003; chi-square test with Monte-Carlo correction). Thus, diagnostic samples of SARS-CoV-2 exhibited complex mutant spectra, which were characterized by a predominance of low-frequency mutations and amino acid substitutions and a remarkable absence of intermediate frequency substitutions that, in contrast, were present in HCV from infected patients.

## 4. Discussion

In the present article, we have initially summarized results on population dynamics of HCV that serve as a counterpart to studies recently initiated with SARS-CoV-2. Evidence from different laboratories indicates that SARS-CoV-2 isolates from infected patients exhibit complex, dynamic, and compartmentalized mutant spectra and that minority mutations in individual isolates, including vaccine-breakthrough cases, can be represented in the consensus sequence of isolates of later epidemic waves [32,33,40,42,43,46,47,85,86,87,88]. Mutant spectra include many low-frequency point mutations and a remarkable number of deletions, presumably generated through recombination events favored by the limited processivity of the coronavirus polymerases [89,90,91,92]. In terms of variation in the course of the epidemiological expansion of the virus, a current estimate of the rate of evolution of SARS-CoV-2 is (1.2 ± 0.6) × 10^−3^ mutations per nucleotide and year (m/n/y), which has been calculated as an average of ten independent measurements (ranging from the following: 9.9 × 10^−4^ to 2.2 × 10^−3^ m/n/y) [93,94,95,96,97,98,99,100,101,102]. This rate of evolution is comparable to that calculated for other RNA viruses [103]. It is not known whether the rate of evolution of SARS-CoV-2 might be influenced by its being in the process of early adaptation to the human host and if the rate might change in the course of propagation of more recent variants of concern. This is a point that warrants further investigation.

Regarding adaptive potential, the available data on intra-host heterogeneity and rate of evolution of consensus sequences of SARS-CoV-2 do not support any fundamental difference with other RNA viruses of smaller genome size, and which are devoid of proofreading-repair activities [104,105]. It is not known whether the Exo N activity in protein nsp14 of SARS-CoV-2—whose absence impairs viral RNA synthesis [106,107]—contributes to lowering the basal error rate of the nsp12 (polymerase)-containing replication complex [90,108,109,110]. According to work with other coronaviruses, it seems likely that the ExoN of SARS-CoV-2 has some effect on template-copying fidelity, although this is still an open question. From quantitative considerations of SARS-CoV-2 and other coronavirus infections, we have suggested that the presence of a proofreading repair function in SARS-CoV-2 may contribute to limiting virus entry into error catastrophe but may not significantly delay the exploration of new variant sequences in the course of the pandemic spread of the virus ([105] and accompanying quantifications in references quoted therein).

The new information on SARS-CoV-2 mutant spectra presented in this article is derived from the extension of the analysis to reach a 0.1% mutant frequency cut-off. The number of different and total mutations increased massively when the mutant frequency cut-off was lowered to 0.1% (Figure 5). Such lowering was justified by the number of clean reads obtained for each of the amplicons analyzed, so that each mutation scored was represented by hundreds of reads (Table S2). The bias in favor of C → T and A → G (over other mutation types) and the high transition to transversion ratio were maintained (and in some cases, accentuated) with the 0.1% relative to the 0.5% cut-off (Table 1 and Figure S2). Moreover, at the amino acid level, there were no significant differences in substitution acceptability, prediction of functional effects, or presence of the substitutions in the “outbreak.info” (enabled by the GISAID database) was noted (Tables S3 and S5). Thus, all evidence supports that at least the great majority of mutations detected at 0.1% cut-off frequency belong to genomes that populate SARS-CoV-2 diagnostic samples (see also Materials and Methods). Specifically, using the 0.1% frequency cut-off, we identified amino acid substitution Q498R, which is typical of the Omicron lineages, in viruses from two patients, one with mild and another with moderate disease, that were infected during the first COVID-19 wave 20 months earlier (Table S3).

A feature that we noted in the SARS-CoV-2 mutant spectra from diagnostic samples is the scarcity of mutations at intermediate frequencies, and an overwhelming abundance of mutations at frequencies lower than 10% [46,47]. The similarity of bioinformatics pipelines used to characterize the SARS-CoV-2 and HCV amino acid spectra has allowed a comparative study with viruses sampled from infected patients. In such a comparison, HCV does not show the deficit of intermediate frequency substitutions that is observed with SARS-CoV-2. This difference was maintained when two functionally equivalent proteins, the corresponding viral polymerases, were compared (Figure 6). The presence of intermediate frequency mutations in HCV was also observed in laboratory populations (HCV p0, HCV p100, and HCV p200, with primary sequencing data described in [59]). In these studies, with a point mutation frequency cut-off of 0.5%, the mutations present at a frequency higher than 10% averaged 19.2%, 37.7%, and 37.6% for HCV p0, HCV p100, and HCV p200, respectively, with percentages consistent in three biological replicas of the experiment (Figure S3). The reason for the difference in the mutation frequency distribution between SARS-CoV-2 and HCV is unknown. The early results of comparison of genomic nucleotide sequences of populations or clones of bacteriophage Qβ, foot-and-mouth disease virus, and lymphocytic choriomeningitis virus (reviewed in [13]) suggested also distribution of minority mutations closer to that of HCV than SARS-CoV-2. There are several differences among these viruses regarding replication mechanism and interaction with their host organisms that may influence the frequency distribution of minority mutations in virus samples. In particular, HCV chronicity may permit the accommodation of mutated genomes with comparable fitness during prolonged viral replication. Other differences between SARS-CoV-2 and HCV that may also affect the frequency distribution of minority mutations are the error rate during nucleotide incorporation, the proportion of positive- and negative-strand RNA, differences in stability of replicating viral RNA, or inefficiency of particle assembly depending on the mutational load in the RNA molecules, among others. In addition, population parameters such as the number and intensity of bottleneck events prior to virus sampling for nucleotide sequence analysis may also play a role. From an evolutionary perspective, one possibility is that the absence of intermediate frequency mutations may reflect the negative effect on viral fitness of mutations that randomly occur in the large coronavirus genome, as compared with the effect of a similar number of mutations per nucleotide introduced in smaller RNA genomes [16,19,79,111]. However, in experimental infections of camels with a human isolate of MERS-CoV, intermediate frequency mutations were present in the mutant spectrum of nasal samples of the animals [88]. This observation suggests that the large size of the coronavirus genome per se is not sufficient to account for the absence of intermediate frequency mutations. Additional work is necessary to clarify this important point since it bears on the adaptive flexibility of coronaviruses.

The number of different and total mutations increased massively when the mutant frequency cut-off was lowered to 0.1% (Figure 5). Such lowering was justified by the number of clean reads obtained for each of the amplicons analyzed, so that each mutation scored was represented by hundreds of reads (Table S2). The bias in favor of C → T and A → G (over other mutation types) and the high transition to transversion ratio were maintained (and in some cases, accentuated) with the 0.1% relative to the 0.5% cut-off (Table 1 and Figure S2). Moreover, at the amino acid level, there were no significant differences in substitution acceptability, prediction of functional effects, or presence of the substitutions in the “outbreak.info” (enabled by the GISAID database) was noted (Tables S3 and S5). Thus, all evidence supports that at least the great majority of mutations detected at 0.1% cut-off frequency belong to genomes that populate SARS-CoV-2 diagnostic samples (see also Materials and Methods). Specifically, using the 0.1% frequency cut-off, we identified amino acid substitution Q498R, which is typical of the Omicron lineages, in viruses from two patients, one with mild and another with moderate disease, that were infected during the first COVID-19 wave 20 months earlier (Table S3).

The extremely broad repertoire of low-frequency mutations in SARS-CoV-2 populations poses a number of intriguing questions, including whether the minority genomes that harbor them are viable or defective. If they are viable, they are likely to belong to genomes that display low fitness at the time of viral sampling. The question is if they can be selected despite their being surrounded by a spectrum of mutants that may suppress them [1]. It will be interesting to investigate if such low-frequency mutations exhibit a mutational wave behavior (change in frequency with time) as described for HCV [58,59]. If this were the case, their frequency would fluctuate, and the carrying genomes would be more prone to becoming dominant as a result of selection or random drift [80]. If, on the contrary, the mutations are present in defective genomes, they may be maintained by complementation [112], and eventually they may become part of viable genomes by recombination [91,92]. The epidemiological data suggest that the same mutations that have low frequency in mutant spectra can be found as dominant in independent isolates. Indeed, 97.6% of the amino acid substitutions that are deduced from the mutations present at the 0.1%–0.49% frequency range (visualized in Figure 2 and Figure 3) are also represented in the consensus sequence of one or several SARS-CoV-2 isolates from different geographical locations and times (indicated in Table S3). Most likely, the same mutations are produced independently in different replicating genomes in a variety of accompanying mutational contexts. It should be pointed out that a 0.1% frequency is still considerably higher than the expected error rate of the coronavirus polymerase (anticipated to lie between 10^−6^ and 10^−4^ mutations introduced per nucleotide copied [31], pending direct measurements with SARS-CoV-2). Independently of the precise moment at which a specific mutation occurs in replicating RNA, the large repertoire identified by ultra-deep sequencing must be located in genomes that have the replicative fitness (by themselves or assisted by complementation) required for detection by current ultra-deep sequencing methods. Additional work is needed to try to elucidate the biological significance and adaptability implications of the enormous reservoir of low-frequency genomes present in SARS-CoV-2 populations.

## Figures and Tables

**Table 1:** Number of different and total mutations in SARS-CoV-2 isolates, classified in mild, moderate, and exitus patients.

				Patient Category		
			Total	Mild	Moderate	Exitus
**nsp12 (polymerase)**	**Number of different mutations**	**Transitions (Ts) (%)**	578 (98.97%)	544 (99.63%)	344 (99.42%)	416 (99.05%)
		**Transversions (Tv) (%)**	6 (1.03%)	2 (0.37%)	2 (0.58%)	4 (0.24%)
		**Ratio (Ts/Tv)**	96.33	272	172	104
		***p*-value**	<0.001	<0.001	<0.001	<0.001
		**Significance ^a^**	***	***	***	***
	**Number of total mutations**	**Transitions (Ts) (%)**	7587 (99.82%)	2883 (99.93%)	2254 (99.65%)	2451 (99.84%)
		**Transversions (Tv) (%)**	14 (0.18%)	2 (0.07%)	8 (0.35%)	4 (0.16%)
		**Ratio (Ts/Tv)**	541.93	1441.50	281.75	612.75
		***p*-value**	<0.001	<0.001	<0.001	<0.001
		**Significance ^a^**	***	***	***	***
**spike**	**Number of different mutations**	**Transitions (Ts) (%)**	297 (99.33%)	273 (100%)	209 (99.52%)	210 (99.53%)
		**Transversions (Tv) (%)**	2 (0.67%)	0 (0%)	1 (0.48%)	1 (0.47%)
		**Ratio (Ts/Tv)**	148.50	-	209	210
		***p*-value**	<0.001	<0.001	<0.001	<0.001
		**Significance ^a^**	***	***	***	***
	**Number of total mutations**	**Transitions (%)**	3718 (99.95%)	1343 (100%)	1187 (99.92%)	1188 (99.92%)
		**Transversions (%)**	2 (0.05%)	0 (0%)	1 (0.08%)	1 (0.08%)
		**Ratio (Ts/Tv)**	1859	-	1187	1188
		***p*-value**	<0.001	<0.001	<0.001	<0.001
		**Significance ^a^**	***	***	***	***

^a^ Statistical difference of significance is given (***, *p* < 0.001).

**Table 2:** Number of different and total mutations in SARS-CoV-2 isolates, classified in mild, moderate and exitus patients.

				Patient Category		
			Total	Mild	Moderate	Exitus
**nsp12 (polymerase)**	**Number of different mutations**	**Synonymous (Syn) (%)**	238 (40.75%)	218 (39.93%)	146 (42.20%)	175 (41.67%)
		**Non-synonymous** **(Non-syn) (%)**	346 (59.25%)	328 (60.07%)	200 (57.80%)	245 (58.33%)
		**Ratio (Syn/Non-syn)**	0.69	0.66	0.73	0.71
		***p*-value**	<0.001	<0.001	<0.001	<0.001
		**Significance ^a^**	***	***	***	***
	**Number of total mutations**	**Synonymous (Syn) (%)**	2971 (39.08%)	1130 (39.17%)	877 (38.78%)	964 (39.27%)
		**Non-synonymous** **(Non-syn) (%)**	4631 (60.92%)	1755 (60.83%)	1385 (61.23%)	1491 (60.73%)
		**Ratio (Syn/Non-syn)**	0.64	0.64	0.63	0.65
		***p*-value**	<0.001	<0.001	<0.001	<0.001
		**Significance ^a^**	***	***	***	***
**spike**	**Number of different mutations**	**Synonymous (Syn) (%)**	125 (41.95%)	115 (42.28%)	90 (43.06%)	89 (42.38%)
		**Non-synonymous** **(Non-syn) (%)**	173 (58.05%)	157 (57.72%)	119 (56.94%)	121 (57.62%)
		**Ratio (Syn/Non-syn)**	0.72	0.73	0.76	0.74
		***p*-value**	<0.001	<0.001	0.006	0.002
		**Significance ^a^**	***	***	***	***
	**Number of total mutations**	**Synonymous (Syn) (%)**	1659 (44.60%)	606 (45.12%)	525 (44.19%)	528 (44.41%)
		**Non-synonymous** **(Non-syn) (%)**	2061 (55.40%)	737 (54.88%)	663 (55.81%)	661 (55.59%)
		**Ratio (Syn/Non-syn)**	0.80	0.82	0.79	0.80
		***p*-value**	<0.001	<0.001	<0.001	<0.001
		**Significance ^a^**	***	***	***	***

^a^ Statistical difference of significance is given (**, *p* < 0.01; ***, *p* < 0.001).

## Data Availability

Fastq files of SARS-CoV-2 samples included in the patient cohort are available in ENA under project id “PRJEB48766” and further details are described in [47]. Nucleotide and amino acid replacements in SARS-CoV-2 from infected patients have been compiled in Table S3.

## Supplementary Materials

The following supporting information can be downloaded at: https://www.mdpi.com/article/10.3390/pathogens11060662/s1, Table S1: Oligonucleotides used to amplify the nsp12 (polymerase)- and spike-coding regions of SARS-CoV-2; Table S2: Number of clean reads obtained in SARS-CoV-2-infected patients with a cut-off of 0.1%; Table S3: Repertoire of point mutations and amino acid substitutions identified in SARS-CoV-2 in nsp12 (polymerase)- and spike-coding regions; Table S4: Deletions found in nsp12 (polymerase)- and spike-coding regions of SARS-CoV-2 isolated from infected patients; Table S5: Statistical analysis of the acceptability of the repertoire of amino acid substitutions identified in nsp12 (polymerase) and spike; Figure S1: Deletion types in the nsp12 (polymerase) and the spike-coding regions; Figure S2: Mutation types in the mutant spectra of SARS-CoV-2; Figure S3: Percentage of total mutations observed in HCV laboratory population (HCV p0, HCV p100, and HCV p200) distributed according to their frequency with a 0.5% as the low frequency limit.
