# Supplementary material for: SARS-CoV-2 Mutant Spectra at Different Depth Levels Reveal an Overwhelming Abundance of Low Frequency Mutations
Source: Pathogens. 2022 Jun 8;11(6):662. doi: 10.3390/pathogens11060662 (PMC9227345; doi:10.3390/pathogens11060662)
Supplement: Supplementary file 1 [file pathogens-11-00662-s001.zip › pathogens-1727479-supplementary.pdf]

Supplementary material for:

SARS-CoV-2 mutant spectra at different depth levels reveal an overwhelming abundance of low frequency mutations

Brenda Martínez-González[1,2], María Eugenia Soria[1,3,4], Lucía Vázquez-Sirvent[1], Cristina Ferrer-Orta[5], Rebeca Lobo-Vega[1], Pablo Mínguez[6,7,8], Lorena de la Fuente[6,7,8], Carlos Llorens[9], Beatriz Soriano[9], Ricardo Ramos-Ruíz[10], Marta Cortón[6,7], Rosario López-Rodríguez[6,7], Carlos García-Crespo[3,4], Pilar Somovilla[3,11], Antoni Durán-Pastor[3], Isabel Gallego[3,4], Ana Isabel de Ávila[3,4], Soledad Delgado[12], Federico Morán[13], Cecilio López-Galíndez[14], Jordi Gómez[4,15], Luis Enjuanes[2], Llanos Salar-Vidal[1], Mario Esteban-Muñoz[1], Jaime Esteban[1], Ricardo Fernandez-Roblas[1], Ignacio Gadea[1], Carmen Ayuso[6,7], Javier Ruíz-Hornillos[16,17,18], Nuria Verdaguer[5], Esteban Domingo[3,4*] and Celia Perales[1,2,4*]

[1]Department of Clinical Microbiology, Instituto de Investigación Sanitaria-Fundación Jiménez Díaz University Hospital, Universidad Autónoma de Madrid (IIS-FJD, UAM) Av. Reyes Católicos 2, 28040 Madrid, Spain, [2]Department of Molecular and Cell Biology, Centro Nacional de Biotecnología (CNB-CSIC), Consejo Superior de Investigaciones Científicas (CSIC), Campus de Cantoblanco, 28049 Madrid, Spain, [3]Centro de Biología Molecular "Severo Ochoa" (CSIC-UAM), Consejo Superior de Investigaciones Científicas (CSIC), Campus de Cantoblanco, 28049 Madrid, Spain, [4]Centro de Investigación Biomédica en Red de Enfermedades Hepáticas y Digestivas (CIBERehd), Instituto de Salud Carlos III, 28029 Madrid, Spain, [5]Structural Biology Department, Institut de Biología Molecular de Barcelona CSIC, 08028 Barcelona, Spain, [6]Department of Genetics & Genomics, Instituto de Investigación Sanitaria-Fundación Jiménez Díaz University Hospital, Universidad Autónoma de Madrid (IIS-FJD, UAM), Av. Reyes Católicos 2, 28040 Madrid, Spain, [7]Centre for Biomedical Network Research on Rare Diseases (CIBERER), Instituto de Salud Carlos III, 28029 Madrid, Spain, [8]Bioinformatics Unit, Instituto de Investigación Sanitaria-Fundación Jiménez Díaz University Hospital, Universidad Autónoma de Madrid (IIS-FJD, UAM), Madrid 28040, Spain, [9]Biotechvana, "Scientific Park", Universidad de Valencia, 46980 Valencia, Spain, [10]Unidad de Genómica, "Scientific Park of Madrid", Campus de Cantoblanco, 28049 Madrid, Spain, [11]Departamento de Biología Molecular, Universidad Autónoma de Madrid, Campus de Cantoblanco, 28049, Madrid, Spain, [12]Departamento de Sistemas Informáticos, Escuela Técnica Superior de Ingeniería de Sistemas Informáticos (ETSISI), Universidad Politécnica de Madrid, Madrid, Spain, [13]Departamento de Bioquímica y Biología Molecular, Universidad Complutense de Madrid, Madrid, Spain, [14]Unidad de Virología Molecular, Laboratorio de Referencia e Investigación en Retrovirus, Centro Nacional de Microbiología, Instituto de Salud Carlos III, Majadahonda, Madrid, Spain, [15]Instituto de Parasitología y Biomedicina 'López-Neyra' (CSIC), Parque Tecnológico Ciencias de la Salud, Armilla, 18016 Granada, Spain, [16]Allergy Unit, Hospital Infanta Elena, Valdemoro, Madrid, Spain; [17]Instituto de Investigación Sanitaria-Fundación Jiménez Díaz University Hospital, Universidad Autónoma de Madrid (IIS-FJD, UAM), Av. Reyes Católicos 2, 28040 Madrid, Spain; [18]Faculty of Medicine, Universidad Francisco de Vitoria, Madrid, Spain

*Corresponding authors: Celia Perales (celia.perales@cnb.csic.es) and Esteban Domingo (edomingo@cbm.csic.es)

Table S1. Oligonucleotides used to amplify the nsp12 (polymerase)- and spike-coding regions of SARS-CoV-2.

Region	Sense	Primer name	Sequence (5'–3')	Genome positions[a]	Tm (ºC)
nsp12	Fw	nsp12-CoV2-u14511	GGATGTAAACTTACATAGCTCTA	14511-14533	49.9
	Rv	nsp12-CoV2-d14942	GATTTGTCTAGGTTGTTGACGA	14942-14921	51.1
	Fw	nsp12-CoV2-u14890	GATGGTGGCTGTATTAATGCT	14890-14910	50.5
	Rv	nsp12-CoV2-d15320	GGGATGGCTCTATCACATTTA	15320-15300	50.5
	Fw	nsp12-CoV2-u15268	GAAAACCCTCACCTTATGGG	15268-15287	51.8
	Rv	nsp12-CoV2-d15717	AGAGAGTATCATCATTGAGAAATG	15717-15694	50.6
	Fw	nsp12-CoV2-u15646	GATGTTGACACAGACTTTGTGAA	15646-15668	51.7
	Rv	nsp12-CoV2-d16075	CAGCATACTCCTGATTAGGAT	16075-16055	50.5
spike	Fw	spk-CoV2-u22853	GGCTGCGTTATAGCTTGGA	22853-22871	51.1
	Rv	spk-CoV2-d23288	CAGCATCAGTAGTGTCAGCA	23288-23269	51.8
	Fw	spk-CoV2-u23236	GTTTCTGCCTTTCCAACAATTTG	23236-23258	51.7
	Rv	spk-CoV2-d23666	CTGCACCAAGTGACATAGTGT	23666-23646	52.4

[a]The SARS-CoV-2 genome residue numbering is according to the NCBI reference sequence (accession number NC_045512.2).

Table S2. Number of clean reads obtained in SARS-CoV-2 infected patients with a cut-off of 0.1%.

	Amplicons						
**Patient**	**A1**	**A2**	**A3**	**A4**	**A5**	**A6**	**Total**
**Pt341**	63,555	39,003	72,981	65,495	82,712	76,115	**399,861**
**Pt342**	82,712	60,165	91,095	85,240	104,974	94,499	**518,685**
**Pt354**	79,940	80,889	111,591	112,259	130,537	110,655	**625,871**
**Pt416**	114,111	112,911	160,777	116,627	nd	142,232	**646,658**
**Pt417**	118,569	69,823	102,383	95,960	130,990	128,259	**645,984**
**Pt426**	150,647	75,589	130,013	170,598	146,732	111,163	**784,742**
**Pt427**	132,011	101,021	172,554	133,988	nd	136,749	**676,323**
**Pt441**	93,828	103,492	98,941	92,164	82,660	112,545	**583,630**
**Pt443**	90,974	79,714	84,655	85,457	89,970	89,580	**520,350**
**Pt448**	91,036	70,524	93,156	88,410	124,340	99,446	**566,912**
**Pt106**	133,442	140,207	155,602	124,104	181,128	160,437	**894,920**
**Pt130**	126,346	128,606	149,404	124,206	172,770	146,875	**848,207**
**Pt138**	125,001	144,501	196,471	110,230	141,596	192,928	**910,727**
**Pt142**	89,514	96,501	114,938	75,965	86,840	104,886	**568,644**
**Pt145**	121,228	133,986	158,181	147,049	189,110	145,894	**895,448**
**Pt148**	114,793	83,090	172,961	133,897	156,290	154,926	**815,957**
**Pt150**	99,291	86,793	118,571	143,375	178,313	161,908	**788,251**
**Pt165**	146,728	109,106	149,414	136,126	215,662	140,709	**897,745**
**Pt168**	140,170	109,016	148,138	147,460	164,144	166,348	**875,276**
**Pt182**	137,787	115,948	167,192	149,942	177,236	154,440	**902,545**
**Pt012**	137,663	115,247	143,248	114,790	172,779	129,763	**813,490**
**Pt013**	93,294	78,621	94,140	91,933	110,291	88,725	**557,004**
**Pt017**	91,865	48,108	90,196	76,281	89,520	88,153	**484,123**
**Pt020**	79,558	72,137	86,046	83,132	116,157	70,143	**507,173**
**Pt023**	87,922	74,407	111,557	91,144	97,039	81,826	**543,895**
**Pt032**	88,004	76,066	91,800	87,811	95,603	91,222	**530,506**
**Pt036**	74,328	64,278	75,479	79,503	nd	82,090	**375,678**
**Pt040**	57,515	43,973	53,626	51,165	60,104	50,844	**317,227**
**Pt045**	101,977	84,026	95,976	97,520	113,687	96,802	**589,988**
**Pt047**	86,472	78,295	89,128	90,025	93,607	80,551	**518,078**
**Average**	**105,009**	**89,201**	**119,340**	**106,729**	**129,807**	**116,357**	

nd: no data

Table S3. Repertoire of point mutations and amino acid substitutions identified in SARS-CoV-2 in nsp12 (polymerase)- and spike-coding regions.

							Number of patients		
Region[a]	Mutation[b]	Type (Ts/Tv)[c]	Substitution[d]	Type (Syn/Non-syn)[e]	PAM250[f]	SNAP2[g]	Mild	Moderate	Exitus
nsp12 A1	T14537C	Ts	L366P	Non-syn	-3	effect (55)	6	4	7
nsp12 A1	T14538C	Ts	L366	Syn			1	0	0
nsp12 A1	T14541C	Ts	S367	Syn			10	10	10
nsp12 A1	T14543C	Ts	F368S	Non-syn	-3	effect (9)	1	0	0
nsp12 A1	T14551C	Ts	L371	Syn			1	0	2
nsp12 A1	C14554T	Ts	L372F	Non-syn	2	effect (8)	0	0	1
nsp12 A1	T14555C	Ts	L372P	Non-syn	-3	effect (45)	10	9	10
nsp12 A1	T14556C	Ts	L372	Syn			1	0	0
nsp12 A1	T14558C	Ts	V373A	Non-syn	0	neutral (-55)	7	1	4
nsp12 A1	T14560C	Ts	Y374H	Non-syn	0	effect (62)	1	0	0
nsp12 A1	T14562C	Ts	Y374	Syn			7	5	5
nsp12 A1	T14565C	Ts	A375	Syn			6	5	6
nsp12 A1	T14568C	Ts	A376	Syn			8	10	9
nsp12 A1	A14570G	Ts	D377G	Non-syn	1	effect (36)	1	0	1
nsp12 A1	T14574C	Ts	P378	Syn			10	8	9
nsp12 A1	C14576T	Ts	A379V	Non-syn	0	effect (9)	1	0	0
nsp12 A1	T14577C	Ts	A379	Syn			2	0	0
nsp12 A1	T14579C	Ts	M380T	Non-syn	-1	neutral (-46)	7	7	8
nsp12 A1	A14582G	Ts	H381R	Non-syn	2	neutral (-57)	3	2	2
nsp12 A1	C14583T	Ts	H381	Syn			1	0	0
nsp12 A1	C14585T	Ts	A382V	Non-syn	0	neutral (-94)	1	0	0
nsp12 A1	T14586C	Ts	A382	Syn			4	1	2
nsp12 A1	T14589C	Ts	A383	Syn			10	10	10
nsp12 A1	T14590C	Ts	S384P	Non-syn	1	effect (21)	10	10	10
nsp12 A1	T14592C	Ts	S384	Syn			5	3	3
nsp12 A1	T14595C	Ts	G385	Syn			2	1	0
nsp12 A1	A14597G	Ts	N386S	Non-syn	1	neutral (-94)	0	1	0
nsp12 A1	T14598C	Ts	N386	Syn			1	0	2
nsp12 A1	T14602C	Ts	L388	Syn			2	0	1
nsp12 A1	T14606C	Ts	L389P	Non-syn	-3	effect (59)	1	0	1
nsp12 A1	A14607G	Ts	L389	Syn			1	0	0
nsp12 A1	A14609G[h]	Ts	D390G	Non-syn	1	effect (14)	0	0	1
nsp12 A1	A14613G	Ts	K391	Syn			2	0	2
nsp12 A1	A14617G	Ts	T393A	Non-syn	1	neutral (-1)	3	1	3
nsp12 A1	T14619C	Ts	T393	Syn			1	0	0

nsp12 A1	T14623C	Ts	C395R	Non-syn	-4	effect (71)	9	8	7
nsp12 A1	T14626C	Ts	F396L	Non-syn	2	neutral (-86)	10	10	10
nsp12 A1	T14627C	Ts	F396S	Non-syn	-3	effect (53)	5	0	4
nsp12 A1	T14628C	Ts	F396	Syn			3	0	2
nsp12 A1	T14629C	Ts	S397P	Non-syn	1	effect (56)	1	0	0
nsp12 A1	A14631G	Ts	S397	Syn			2	0	1
nsp12 A1	T14633C	Ts	V398A	Non-syn	0	neutral (-73)	2	0	1
nsp12 A1	T14637C	Ts	A399	Syn			10	10	10
nsp12 A1	T14642C	Ts	L401P	Non-syn	-3	effect (20)	6	5	6
nsp12 A1	T14646C	Ts	T402	Syn			4	0	1
nsp12 A1	A14647G	Ts	N403D	Non-syn	2	neutral (-88)	1	0	0
nsp12 A1	A14648G	Ts	N403S	Non-syn	1	neutral (-91)	4	2	1
nsp12 A1	T14652C	Ts	N404	Syn			1	0	0
nsp12 A1	T14654C	Ts	V405A	Non-syn	0	neutral (-57)	10	10	10
nsp12 A1	T14655C	Ts	V405	Syn			5	3	4
nsp12 A1	T14658C	Ts	A406	Syn			10	10	10
nsp12 A1	T14659C	Ts	F407L	Non-syn	2	neutral (-81)	8	9	9
nsp12 A1	T14660C	Ts	F407S	Non-syn	-3	neutral (-40)	1	0	1
nsp12 A1	T14661C	Ts	F407	Syn			0	0	1
nsp12 A1	A14663G	Ts	Q408R	Non-syn	1	effect (4)	2	0	0
nsp12 A1	A14665G	Ts	T409A	Non-syn	1	neutral (-56)	8	7	9
nsp12 A1	T14667C	Ts	T409	Syn			3	0	2
nsp12 A1	T14669C	Ts	V410A	Non-syn	0	neutral (-45)	10	10	10
nsp12 A1	A14671G	Ts	K411E	Non-syn	0	effect (44)	7	5	5
nsp12 A1	A14672G	Ts	K411R	Non-syn	3	neutral (-87)	1	0	2
nsp12 A1	A14673G	Ts	K411	Syn			10	10	10
nsp12 A1	C14676T	Ts	P412	Syn			1	0	0
nsp12 A1	T14679C	Ts	G413	Syn			1	0	0
nsp12 A1	A14687G	Ts	N416S	Non-syn	1	neutral (-39)	4	1	5
nsp12 A1	A14691G	Ts	K417	Syn			9	9	10
nsp12 A1	A14693G	Ts	D418G	Non-syn	1	effect (9)	8	10	10
nsp12 A1	C14694T	Ts	D418	Syn			1	0	0
nsp12 A1	T14695C	Ts	F419L	Non-syn	2	effect (31)	10	10	10
nsp12 A1	T14700C	Ts	Y420	Syn			6	3	4
nsp12 A1	A14702G	Ts	D421G	Non-syn	1	neutral (-7)	10	10	10
nsp12 A1	T14704C	Ts	F422L	Non-syn	2	neutral (-25)	10	10	10
nsp12 A1	T14709C	Ts	A423	Syn			10	10	10
nsp12 A1	T14711C	Ts	V424A	Non-syn	0	neutral (-69)	10	10	10
nsp12 A1	T14713C	Ts	S425P	Non-syn	1	neutral (-36)	10	10	10
nsp12 A1	A14717G	Ts	K426R	Non-syn	3	neutral (-93)	8	5	7
nsp12 A1	T14721C	Ts	G427	Syn			10	10	10

nsp12 A1	T14722C	Ts	F428L	Non-syn	2	neutral (-85)	2	0	2
nsp12 A1	T14723C	Ts	F428S	Non-syn	-3	effect (21)	3	0	2
nsp12 A1	C14724T	Ts	F428	Syn			1	0	0
nsp12 A1	T14725C	Ts	F429L	Non-syn	2	neutral (-94)	5	0	2
nsp12 A1	A14729G	Ts	K430R	Non-syn	3	neutral (-84)	10	10	10
nsp12 A1	A14732G	Ts	E431G	Non-syn	0	effect (15)	4	1	4
nsp12 A1	A14733G	Ts	E431	Syn			10	10	10
nsp12 A1	A14736G	Ts	G432	Syn			1	0	2
nsp12 A1	A14737G	Ts	S433G	Non-syn	1	neutral (-79)	10	10	10
nsp12 A1	T14739C	Ts	S433	Syn			10	10	9
nsp12 A1	T14740C	Ts	S434P	Non-syn	1	neutral (-82)	8	10	9
nsp12 A1	T14742C	Ts	S434	Syn			2	0	0
nsp12 A1	T14744C	Ts	V435A	Non-syn	0	neutral (-25)	4	0	2
nsp12 A1	T14745C	Ts	V435	Syn			4	0	1
nsp12 A1	A14747G	Ts	E436G	Non-syn	0	neutral (-64)	1	0	2
nsp12 A1	A14754G	Ts	K438	Syn	0		10	10	10
nsp12 A1	A14756G	Ts	H439R	Non-syn	2	effect (56)	4	1	3
nsp12 A1	T14758C	Ts	F440L	Non-syn	2	effect (26)	10	10	10
nsp12 A1	T14759C	Ts	F440S	Non-syn	-3	effect (35)	2	0	2
nsp12 A1	T14761C	Ts	F441L	Non-syn	2	effect (42)	10	10	10
nsp12 A1	T14762C	Ts	F441S	Non-syn	-3	effect (56)	1	0	0
nsp12 A1	T14764C	Ts	F442L	Non-syn	2	effect (37)	8	5	5
nsp12 A1	T14765C	Ts	F442S	Non-syn	-3	effect (26)	1	0	0
nsp12 A1	T14766C	Ts	F442	Syn			1	0	0
nsp12 A1	T14769C	Ts	A443	Syn			10	9	10
nsp12 A1	A14771G	Ts	Q444R	Non-syn	1	effect (19)	9	6	5
nsp12 A1	A14774G	Ts	D445G	Non-syn	1	effect (20)	10	10	10
nsp12 A1	T14775C	Ts	D445	Syn			2	0	0
nsp12 A1	T14778C	Ts	G446	Syn			0	0	1
nsp12 A1	A14780G	Ts	N447S	Non-syn	1	neutral (-92)	3	0	1
nsp12 A1	T14784C	Ts	A448	Syn			5	5	4
nsp12 A1	C14786T	Ts	A449V	Non-syn	0	neutral (-21)	1	0	0
nsp12 A1	T14789C	Ts	I450T	Non-syn	0	neutral (-46)	4	1	3
nsp12 A1	A14791G	Ts	S451G	Non-syn	1	neutral (-47)	8	6	5
nsp12 A1	T14796C	Ts	D452	Syn			3	0	1
nsp12 A1	A14801G	Ts	D454G	Non-syn	1	effect (20)	10	10	10
nsp12 A1	A14804G	Ts	Y455C	Non-syn	0	effect (27)	1	0	0
nsp12 A1	C14805T[i]	Ts	Y455	Syn			2	2	1
nsp12 A1	T14808C	Ts	Y456	Syn			3	0	3
nsp12 A1	T14811C	Ts	R457	Syn			4	1	2
nsp12 A1	T14814C	Ts	Y458	Syn			1	0	0

nsp12 A1	A14816G	Ts	N459S	Non-syn	1	neutral (-22)	1	0	0
nsp12 A1	T14817C	Ts	N459	Syn			1	0	1
nsp12 A1	A14820G	Ts	L460	Syn			2	0	0
nsp12 A1	A14823G	Ts	P461	Syn			7	7	7
nsp12 A1	A14824G	Ts	T462A	Non-syn	1	neutral (-41)	7	5	5
nsp12 A1	A14826G	Ts	T462	Syn			1	0	0
nsp12 A1	A14827G	Ts	M463V	Non-syn	2	neutral (-27)	1	0	1
nsp12 A1	T14828C	Ts	M463T	Non-syn	-1	effect (63)	3	0	0
nsp12 A1	T14830C	Ts	C464R	Non-syn	-4	effect (61)	7	7	8
nsp12 A1	T14832C	Ts	C464	Syn			5	2	5
nsp12 A1	A14834G	Ts	D465G	Non-syn	1	effect (28)	10	10	10
nsp12 A1	A14836G	Ts	I466V	Non-syn	4	neutral (-71)	2	0	0
nsp12 A1	T14837C	Ts	I466T	Non-syn	0	effect (5)	5	1	4
nsp12 A1	A14839G	Ts	R467G	Non-syn	-3	neutral (-15)	8	7	5
nsp12 A1	A14841G	Ts	R467	Syn			10	10	10
nsp12 A1	A14843G	Ts	Q468R	Non-syn	1	effect (30)	4	2	6
nsp12 A1	A14844G	Ts	Q468	Syn			6	8	8
nsp12 A1	A14847G	Ts	L469	Syn			0	0	1
nsp12 A1	T14851C	Ts	F471L	Non-syn	2	effect (2)	1	0	0
nsp12 A1	T14851G	Tv	F471V	Non-syn	-1	neutral (-54)	0	4	1
nsp12 A1	T14853C	Ts	F471	Syn			1	0	0
nsp12 A1	T14855A	Tv	V472E	Non-syn	-2	neutral (-36)	0	4	1
nsp12 A1	T14858C	Ts	V473A	Non-syn	0	neutral (-52)	2	0	0
nsp12 A1	T14859C	Ts	V473	Syn			1	0	0
nsp12 A1	A14861G	Ts	E474G	Non-syn	0	neutral (-7)	2	0	2
nsp12 A1	A14862G	Ts	E474	Syn			9	5	7
nsp12 A1	T14864C	Ts	V475A	Non-syn	0	neutral (-23)	2	0	0
nsp12 A1	T14867C	Ts	V476A	Non-syn	0	neutral (-54)	3	0	2
nsp12 A1	T14868C	Ts	V476	Syn			0	0	2
nsp12 A1	A14870G	Ts	D477G	Non-syn	1	neutral (-30)	9	3	5
nsp12 A1	A14872G	Ts	K478E	Non-syn	0	neutral (-51)	1	0	0
nsp12 A1	A14873G	Ts	K478R	Non-syn	3	neutral (-77)	10	7	7
nsp12 A1	A14876G	Ts	Y479C	Non-syn	0	effect (11)	1	0	0
nsp12 A1	T14878C	Ts	F480L	Non-syn	2	neutral (-47)	4	1	1
nsp12 A1	T14879C	Ts	F480S	Non-syn	-3	effect (34)	1	0	0
nsp12 A1	A14882G	Ts	D481G	Non-syn	1	neutral (-54)	0	0	1
nsp12 A1	T14886C	Ts	C482	Syn			0	0	2
nsp12 A1	A14888G	Ts	Y483C	Non-syn	0	effect (42)	2	0	1
nsp12 A1	A14891G	Ts	D484G	Non-syn	1	effect (42)	10	10	10
nsp12 A1	T14892C	Ts	D484	Syn			1	0	0
nsp12 A1	C14898T	Ts	G486	Syn			0	1	0

nsp12 A1	T14899C	Ts	C487R	Non-syn	-4	neutral (-37)	5	0	1
nsp12 A1	T14903C	Ts	I488T	Non-syn	0	effect (2)	2	0	0
nsp12 A1	A14905G	Ts	N489D	Non-syn	2	neutral (-77)	1	0	0
nsp12 A1	A14906G	Ts	N489S	Non-syn	1	neutral (-97)	1	0	0
nsp12 A1	A14911G	Ts	N491D	Non-syn	2	neutral (-72)	3	1	1
nsp12 A1	A14912G	Ts	N491S	Non-syn	1	neutral (-89)	1	1	1
nsp12 A1/A2	C14914A	Tv	Q492K	Non-syn	1	neutral (-10)	0	0	1
nsp12 A1/A2	A14916G	Ts	Q492	Syn			10	7	9
nsp12 A2	T14918C	Ts	V493A	Non-syn	0	neutral (-12)	10	10	10
nsp12 A1	A14920G	Ts	I494V	Non-syn	4	neutral (-96)	5	3	7
nsp12 A2	T14921C	Ts	I494T	Non-syn	0	neutral (-13)	4	1	2
nsp12 A2	G14923A	Ts	V495I	Non-syn	4	neutral (-80)	1	0	0
nsp12 A2	T14924C	Ts	V495A	Non-syn	0	effect (1)	10	10	10
nsp12 A2	C14925T	Ts	V495	Syn			0	1	0
nsp12 A2	A14930G	Ts	N497S	Non-syn	1	effect (6)	3	0	1
nsp12 A2	C14931T	Ts	N497	Syn			0	0	1
nsp12 A2	A14936G	Ts	D499G	Non-syn	1	effect (70)	6	5	5
nsp12 A2	T14946C	Ts	A502	Syn			6	6	5
nsp12 A2	T14949C	Ts	G503	Syn			10	10	10
nsp12 A2	T14950C	Ts	F504L	Non-syn	2	effect (30)	5	6	2
nsp12 A2	T14951C	Ts	F504S	Non-syn	-3	effect (59)	10	9	8
nsp12 A2	A14964G	Ts	K508	Syn			2	0	1
nsp12 A2	T14976C	Ts	A512	Syn			7	6	7
nsp12 A2	A14979G	Ts	R513	Syn			4	1	2
nsp12 A2	T14981C	Ts	L514P	Non-syn	-3	neutral (-34)	10	9	9
nsp12 A2	T14985C	Ts	Y515	Syn			1	0	0
nsp12 A2	T14986C	Ts	Y516H	Non-syn	0	effect (57)	1	0	0
nsp12 A2	T14988C	Ts	Y516	Syn			3	0	0
nsp12 A2	A14990G	Ts	D517G	Non-syn	1	effect (46)	1	0	0
nsp12 A2	T14992C	Ts	S518P	Non-syn	1	effect (3)	1	0	0
nsp12 A2	A14998G	Ts	S520G	Non-syn	1	neutral (-80)	7	2	8
nsp12 A2	T15000C	Ts	S520	Syn			9	10	10
nsp12 A2	T15003C	Ts	Y521	Syn			1	0	0
nsp12 A2	G15004A	Ts	E522K	Non-syn	0	neutral (-55)	1	0	0
nsp12 A2	A15005G	Ts	E522G	Non-syn	0	neutral (-18)	10	8	10
nsp12 A2	T15009C	Ts	D523	Syn			9	10	9
nsp12 A2	A15012G	Ts	Q524	Syn			7	3	0
nsp12 A2	A15014G	Ts	D525G	Non-syn	1	effect (26)	9	10	9
nsp12 A2	T15015C	Ts	D525	Syn			8	8	9
nsp12 A2	C15017T	Ts	A526V	Non-syn	0	neutral (-36)	0	0	2
nsp12 A2	A15018G	Ts	A526	Syn			5	6	6

nsp12 A2	T15020C	Ts	L527P	Non-syn	-3	effect (74)	9	10	10
nsp12 A2	T15020A[h]	Tv	L527H	Non-syn	-2	effect (51)	1	0	0
nsp12 A2	T15022C	Ts	F528L	Non-syn	2	effect (23)	1	0	0
nsp12 A2	C15024T	Ts	F528	Syn			0	0	1
nsp12 A2	A15027G	Ts	A529	Syn			3	0	1
nsp12 A2	A15034G	Ts	K532E	Non-syn	0	effect (51)	1	0	0
nsp12 A2	A15036G	Ts	K532	Syn			9	9	8
nsp12 A2	T15044C	Ts	V535A	Non-syn	0	effect (14)	10	10	10
nsp12 A2	A15046G	Ts	I536V	Non-syn	4	neutral (-27)	3	0	5
nsp12 A2	T15047C	Ts	I536T	Non-syn	0	neutral (-20)	8	7	2
nsp12 A2	T15051C	Ts	P537	Syn			1	1	1
nsp12 A2	A15058G[h]	Ts	T540A	Non-syn	1	effect (27)	2	0	0
nsp12 A2	T15060C	Ts	T540	Syn			9	9	8
nsp12 A2	T15069C	Ts	N543	Syn			5	0	1
nsp12 A2	T15071C	Ts	L544P	Non-syn	-3	effect (46)	1	0	0
nsp12 A2	A15074G	Ts	K545R	Non-syn	3	effect (36)	7	2	4
nsp12 A2	T15078C	Ts	Y546	Syn			6	4	6
nsp12 A2	A15082G	Ts	I548V	Non-syn	4	neutral (-35)	1	0	0
nsp12 A2	T15083C	Ts	I548T	Non-syn	0	effect (15)	1	0	0
nsp12 A2	T15087C	Ts	S549	Syn			1	0	0
nsp12 A2	A15090G	Ts	A550	Syn			5	1	2
nsp12 A2	A15092G	Ts	K551R	Non-syn	3	neutral (-25)	1	0	0
nsp12 A2	A15095G	Ts	N552S	Non-syn	1	neutral (-94)	10	10	10
nsp12 A2	A15099G	Ts	R553	Syn			10	10	10
nsp12 A2	T15102C	Ts	A554	Syn			10	10	9
nsp12 A2	C15103T	Ts	R555C	Non-syn	-4	effect (68)	1	0	0
nsp12 A2	A15106G	Ts	T556A	Non-syn	1	effect (57)	6	0	2
nsp12 A2	C15107T	Ts	T556I	Non-syn	0	effect (73)	1	0	0
nsp12 A2	G15109A	Ts	V557I	Non-syn	4	neutral (-51)	0	0	1
nsp12 A2	T15110C	Ts	V557A	Non-syn	0	effect (22)	0	0	1
nsp12 A2	A15111G	Ts	V557	Syn			1	0	0
nsp12 A2	T15114C	Ts	A558	Syn			4	5	4
nsp12 A2	T15117C	Ts	G559	Syn			10	8	9
nsp12 A2	T15119C	Ts	V560A	Non-syn	0	effect (3)	10	10	10
nsp12 A2	T15121C	Ts	S561P	Non-syn	1	effect (68)	10	10	10
nsp12 A2	T15125C	Ts	I562T	Non-syn	0	effect (20)	10	9	9
nsp12 A2	T15127C	Ts	C563R	Non-syn	-4	effect (78)	9	8	7
nsp12 A2	T15129C	Ts	C563	Syn			0	0	1
nsp12 A2	T15135C	Ts	T565	Syn			2	0	0
nsp12 A2	A15136G	Ts	M566V	Non-syn	2	effect (22)	2	0	0
nsp12 A2	T15137C	Ts	M566T	Non-syn	-1	effect (4)	4	0	1

nsp12 A2	A15139G	Ts	T567A	Non-syn	1	neutral (-28)	10	10	10
nsp12 A2	A15143G	Ts	N568S	Non-syn	1	neutral (-49)	5	3	6
nsp12 A2	A15145G	Ts	R569G	Non-syn	-3	effect (79)	3	0	0
nsp12 A2	A15147G	Ts	R569	Syn			10	10	10
nsp12 A2	A15149G	Ts	Q570R	Non-syn	1	neutral (-56)	1	0	0
nsp12 A2	T15151C	Ts	F571L	Non-syn	2	neutral (-34)	10	10	10
nsp12 A2	T15152C	Ts	F571S	Non-syn	-3	effect (51)	10	10	10
nsp12 A2	T15153C	Ts	F571	Syn			7	4	5
nsp12 A2	T15156C	Ts	H572	Syn			4	2	5
nsp12 A2	A15169G	Ts	K577E	Non-syn	0	effect (45)	1	0	0
nsp12 A2	A15171G	Ts	K577	Syn			7	7	6
nsp12 A2	A15175G	Ts	I579V	Non-syn	4	neutral (0)	9	9	10
nsp12 A2	A15177G	Ts	I579M	Non-syn	2	neutral (-64)	0	0	1
nsp12 A2	A15184G	Ts	T582A	Non-syn	1	neutral (-60)	8	6	5
nsp12 A2	A15187G	Ts	R583G	Non-syn	-3	effect (81)	2	1	0
nsp12 A2	A15189G	Ts	R583	Syn			10	10	10
nsp12 A2	A15192G	Ts	G584	Syn			10	10	10
nsp12 A2	T15195C	Ts	A585	Syn			1	0	0
nsp12 A2	A15196G	Ts	T586A	Non-syn	1	neutral (-39)	1	0	0
nsp12 A2	T15198C	Ts	T586	Syn			8	5	6
nsp12 A2	A15210G	Ts	G590	Syn			2	0	0
nsp12 A2	A15211G	Ts	T591A	Non-syn	1	effect (34)	10	10	10
nsp12 A2	A15214G	Ts	S592G	Non-syn	1	effect (54)	9	9	7
nsp12 A2	C15216T	Ts	S592	Syn			0	1	0
nsp12 A2	A15218G	Ts	K593R	Non-syn	3	effect (57)	1	0	0
nsp12 A2	T15228C	Ts	G596	Syn			1	0	0
nsp12 A2	T15231C	Ts	G597	Syn			1	0	0
nsp12 A2	T15232C	Ts	W598R	Non-syn	2	effect (90)	2	1	2
nsp12 A2	A15236G	Ts	H599R	Non-syn	2	effect (63)	10	8	7
nsp12 A2	C15237T	Ts	H599	Syn			0	0	1
nsp12 A2	A15238G	Ts	N600D	Non-syn	2	neutral (-64)	4	0	4
nsp12 A2	A15239G	Ts	N600S	Non-syn	1	neutral (-86)	10	10	10
nsp12 A2	C15240T	Ts	N600	Syn			0	1	1
nsp12 A2	A15241G	Ts	M601V	Non-syn	2	neutral (-27)	8	4	5
nsp12 A2	T15242C	Ts	M601T	Non-syn	-1	neutral (-10)	1	0	0
nsp12 A2	T15244C	Ts	L602	Syn			3	0	1
nsp12 A2	A15250G	Ts	T604A	Non-syn	1	neutral (-62)	9	6	9
nsp12 A2	C15251T	Ts	T604I	Non-syn	0	effect (4)	0	0	1
nsp12 A2	T15254C	Ts	V605A	Non-syn	0	neutral (-40)	6	2	1
nsp12 A2	T15255C	Ts	V605	Syn			1	0	0
nsp12 A2	A15259G	Ts	S607G	Non-syn	1	neutral (-92)	1	0	0

nsp12 A2	T15261C	Ts	S607	Syn			2	0	0
nsp12 A2	A15263G	Ts	D608G	Non-syn	1	neutral (-83)	10	10	10
nsp12 A2	A15267G	Ts	V609	Syn			4	0	3
nsp12 A2	A15270G	Ts	E610	Syn			10	10	10
nsp12 A2	A15271G	Ts	N611D	Non-syn	2	neutral (-63)	3	0	0
nsp12 A2	A15272G	Ts	N611S	Non-syn	1	neutral (-49)	10	10	10
nsp12 A2	T15276C	Ts	P612	Syn			5	0	2
nsp12 A2	A15278G	Ts	H613R	Non-syn	2	neutral (-76)	8	10	5
nsp12 A2	T15281C	Ts	L614P	Non-syn	-3	effect (48)	1	0	0
nsp12 A3	T15288C	Ts	G616	Syn			10	10	10
nsp12 A3	G15292A	Ts	D618N	Non-syn	2	effect (75)	1	0	0
nsp12 A3	T15294C	Ts	D618	Syn			6	1	5
nsp12 A3	T15297C	Ts	Y619	Syn			9	10	9
nsp12 A3	T15306C	Ts	C622	Syn			10	8	8
nsp12 A3	A15308G	Ts	D623G	Non-syn	1	effect (75)	2	1	4
nsp12 A3	A15312G	Ts	R624	Syn			9	9	9
nsp12 A3	T15317C	Ts	M626T	Non-syn	-1	effect (44)	7	6	6
nsp12 A3	T15321C	Ts	P627	Syn			5	5	5
nsp12 A3	A15323G	Ts	N628S	Non-syn	1	neutral (-35)	3	0	0
nsp12 A3	A15325G	Ts	M629V	Non-syn	2	neutral (-79)	1	0	0
nsp12 A3	T15329C	Ts	L630P	Non-syn	-3	effect (61)	10	9	7
nsp12 A3	T15343C	Ts	S635P	Non-syn	1	effect (27)	10	10	10
nsp12 A3	T15347C	Ts	L636P	Non-syn	-3	effect (39)	10	10	10
nsp12 A3	T15348C	Ts	L636	Syn			1	0	0
nsp12 A3	T15350C	Ts	V637A	Non-syn	0	neutral (-7)	10	10	10
nsp12 A3	T15351C	Ts	V637	Syn			10	10	10
nsp12 A3	T15353C	Ts	L638P	Non-syn	-3	effect (67)	10	10	10
nsp12 A3	T15357C	Ts	A639	Syn			10	10	10
nsp12 A3	C15358T	Ts	R640C	Non-syn	-4	effect (56)	1	0	0
nsp12 A3	A15363G	Ts	K641	Syn			1	1	1
nsp12 A3	A15365G	Ts	H642R	Non-syn	2	effect (29)	1	0	0
nsp12 A3	T15366C	Ts	H642	Syn			0	0	1
nsp12 A3	A15369G	Ts	T643	Syn			5	4	5
nsp12 A3	A15370G	Ts	T644A	Non-syn	1	neutral (-63)	9	7	8
nsp12 A3	T15373C	Ts	C645R	Non-syn	-4	effect (69)	7	6	6
nsp12 A3	T15375C	Ts	C645	Syn			10	10	10
nsp12 A3	T15376C	Ts	C646R	Non-syn	-4	effect (47)	7	5	6
nsp12 A3	T15378C	Ts	C646	Syn			1	1	1
nsp12 A3	T15382C	Ts	L648	Syn			10	10	10
nsp12 A3	T15383C	Ts	L648S	Non-syn	-3	neutral (-73)	10	10	10
nsp12 A3	T15385C	Ts	S649P	Non-syn	1	neutral (-59)	10	10	10

nsp12 A3	A15387G	Ts	S649	Syn			8	8	8
nsp12 A3	A15389G	Ts	H650R	Non-syn	2	effect (7)	9	9	10
nsp12 A3	T15393C	Ts	R651	Syn			10	10	10
nsp12 A3	T15394C	Ts	F652L	Non-syn	2	neutral (-43)	9	10	10
nsp12 A3	T15395C	Ts	F652S	Non-syn	-3	neutral (-11)	1	0	0
nsp12 A3	T15397C	Ts	Y653H	Non-syn	0	effect (23)	2	2	0
nsp12 A3	A15405G	Ts	L655	Syn			1	0	0
nsp12 A3	T15408C	Ts	A656	Syn			5	8	3
nsp12 A3	A15413G	Ts	E658G	Non-syn	0	effect (62)	10	10	9
nsp12 A3	T15415C	Ts	C659R	Non-syn	-4	effect (63)	10	10	9
nsp12 A3	T15417C	Ts	C659	Syn			8	6	9
nsp12 A3	C15419T	Ts	A660V	Non-syn	0	neutral (-29)	1	0	0
nsp12 A3	T15420C	Ts	A660	Syn			10	10	10
nsp12 A3	A15423G	Ts	Q661	Syn			6	5	4
nsp12 A3	T15425C	Ts	V662A	Non-syn	0	effect (16)	1	0	0
nsp12 A3	A15430G	Ts	S664G	Non-syn	1	effect (6)	8	8	7
nsp12 A3	T15432C	Ts	S664	Syn			1	0	0
nsp12 A3	A15434G	Ts	E665G	Non-syn	0	effect (58)	10	10	9
nsp12 A3	A15435G	Ts	E665	Syn			1	0	0
nsp12 A3	A15436G	Ts	M666V	Non-syn	2	neutral (-78)	10	10	10
nsp12 A3	G15439A	Ts	V667I	Non-syn	4	effect (32)	1	0	0
nsp12 A3	T15440C	Ts	V667A	Non-syn	0	neutral (-19)	10	10	10
nsp12 A3	A15442G	Ts	M668V	Non-syn	2	neutral (-54)	3	0	1
nsp12 A3	T15443C	Ts	M668T	Non-syn	-1	neutral (-52)	7	8	9
nsp12 A3	T15445C	Ts	C669R	Non-syn	-4	effect (50)	10	10	9
nsp12 A3	T15447C	Ts	C669	Syn			9	8	9
nsp12 A3	T15453C	Ts	G671	Syn			10	10	10
nsp12 A3	T15454C	Ts	S672P	Non-syn	1	neutral (-2)	8	6	6
nsp12 A3	A15456G	Ts	S672	Syn			7	3	4
nsp12 A3	T15458C	Ts	L673P	Non-syn	-3	effect (74)	2	0	0
nsp12 A3	T15460C	Ts	Y674H	Non-syn	0	effect (71)	1	0	0
nsp12 A3	T15462C	Ts	Y674	Syn			1	0	0
nsp12 A3	T15464C	Ts	V675A	Non-syn	0	neutral (-34)	3	0	1
nsp12 A3	A15468G	Ts	K676	Syn			6	3	4
nsp12 A3	A15471G	Ts	P677	Syn			4	1	1
nsp12 A3	T15474C	Ts	G678	Syn			7	6	7
nsp12 A3	A15477G	Ts	G679	Syn			3	2	0
nsp12 A3	A15478G	Ts	T680A	Non-syn	1	effect (54)	10	10	10
nsp12 A3	T15481C	Ts	S681P	Non-syn	1	effect (72)	10	10	10
nsp12 A3	A15483G	Ts	S681	Syn			4	1	2
nsp12 A3	T15484C	Ts	S682P	Non-syn	1	effect (83)	8	6	5

nsp12 A3	A15486G	Ts	S682	Syn			6	7	7
nsp12 A3	A15489G	Ts	G683	Syn			10	10	10
nsp12 A3	A15491G	Ts	D684G	Non-syn	1	effect (58)	10	10	10
nsp12 A3	T15492C	Ts	D684	Syn			3	0	1
nsp12 A3	A15496G	Ts	T686A	Non-syn	1	effect (41)	7	4	7
nsp12 A3	A15498G	Ts	T686	Syn			2	0	0
nsp12 A3	A15499G	Ts	T687A	Non-syn	1	effect (81)	3	0	1
nsp12 A3	C15503T	Ts	A688V	Non-syn	0	effect (30)	1	0	0
nsp12 A3	T15504C	Ts	A688	Syn			9	6	8
nsp12 A3	A15506G	Ts	Y689C	Non-syn	0	effect (69)	1	0	0
nsp12 A3	T15507C	Ts	Y689	Syn			4	0	3
nsp12 A3	T15510C	Ts	A690	Syn			1	0	0
nsp12 A3	T15516C	Ts	S692	Syn			2	1	0
nsp12 A3	T15518C	Ts	V693A	Non-syn	0	neutral (-41)	9	7	7
nsp12 A3	A15524G	Ts	N695S	Non-syn	1	effect (16)	9	6	8
nsp12 A3	T15527C	Ts	I696T	Non-syn	0	neutral (-7)	1	0	1
nsp12 A3	T15531C	Ts	C697	Syn			10	10	10
nsp12 A3	A15533G[h]	Ts	Q698R	Non-syn	1	effect (63)	9	10	10
nsp12 A3	A15534G	Ts	Q698	Syn			9	10	10
nsp12 A3	T15537C	Ts	A699	Syn			9	10	9
nsp12 A3	T15539C	Ts	V700A	Non-syn	0	neutral (-17)	10	10	10
nsp12 A3	C15540T	Ts	V700	Syn			0	1	0
nsp12 A3	A15541G	Ts	T701A	Non-syn	1	neutral (-27)	10	10	9
nsp12 A3	A15547G	Ts	N703D	Non-syn	2	effect (39)	1	0	0
nsp12 A3	T15551C	Ts	V704A	Non-syn	0	effect (4)	1	0	0
nsp12 A3	A15554G	Ts	N705S	Non-syn	1	neutral (-71)	3	0	2
nsp12 A3	T15555C	Ts	N705	Syn			2	0	0
nsp12 A3	A15558G	Ts	A706	Syn			8	5	5
nsp12 A3	T15560C	Ts	L707P	Non-syn	-3	effect (86)	10	10	10
nsp12 A3	T15562C	Ts	L708	Syn			2	0	0
nsp12 A3	T15565C	Ts	S709P	Non-syn	1	effect (39)	9	8	7
nsp12 A3	T15567C	Ts	S709	Syn			1	0	0
nsp12 A3	T15570C	Ts	T710	Syn			2	0	0
nsp12 A3	G15571A	Ts	D711H	Non-syn	1	neutral (-56)	1	0	0
nsp12 A3	A15572G	Ts	D711G	Non-syn	1	neutral (-55)	9	10	6
nsp12 A3	T15576C	Ts	G712	Syn			1	0	1
nsp12 A3	A15578G	Ts	N713S	Non-syn	1	neutral (-85)	9	10	9
nsp12 A3	A15581G	Ts	K714R	Non-syn	3	neutral (-81)	1	0	0
nsp12 A3	A15582G	Ts	K714	Syn			0	0	1
nsp12 A3	A15583G	Ts	I715V	Non-syn	4	neutral (-85)	5	3	3
nsp12 A3	A15593G	Ts	K718R	Non-syn	3	neutral (-88)	8	5	4

nsp12 A3	A15596G	Ts	Y719C	Non-syn	0	neutral (-6)	1	0	0
nsp12 A3	T15599C	Ts	V720A	Non-syn	0	neutral (-13)	10	6	8
nsp12 A3	A15611G	Ts	Q724R	Non-syn	1	effect (20)	4	0	1
nsp12 A3	A15612G	Ts	Q724	Syn			10	10	10
nsp12 A3	C15613T	Ts	H725Y	Non-syn	0	neutral (-79)	1	0	0
nsp12 A3	A15614G	Ts	H725R	Non-syn	2	neutral (-89)	10	10	9
nsp12 A3	A15616G	Ts	R726G	Non-syn	-3	neutral (-53)	5	1	2
nsp12 A3	A15618G	Ts	R726	Syn			10	10	10
nsp12 A3	T15620C	Ts	L727P	Non-syn	-3	effect (66)	8	9	9
nsp12 A3	T15622C	Ts	Y728H	Non-syn	0	effect (47)	1	0	0
nsp12 A3	A15626G	Ts	E729G	Non-syn	0	neutral (-16)	10	10	10
nsp12 A3	T15628C	Ts	C730R	Non-syn	-4	neutral (-22)	7	1	1
nsp12 A3	T15630C	Ts	C730	Syn			9	8	9
nsp12 A3	T15632C	Ts	L731P	Non-syn	-3	effect (67)	7	8	7
nsp12 A3	A15637G[h]	Ts	R733G	Non-syn	-3	effect (73)	2	1	1
nsp12 A3	A15641G	Ts	N734S	Non-syn	1	neutral (-96)	1	0	0
nsp12 A3	A15645G	Ts	R735	Syn			10	10	10
nsp12 A3	A15647G	Ts	D736G	Non-syn	1	neutral (-52)	9	10	10
nsp12 A3	T15650C	Ts	V737A	Non-syn	0	neutral (-73)	2	0	1
nsp12 A3	T15651C	Ts	V737	Syn			4	1	1
nsp12 A3	A15653G	Ts	D738G	Non-syn	1	effect (36)	10	10	10
nsp12 A3	A15655G	Ts	T739A	Non-syn	1	neutral (-86)	10	10	10
nsp12 A3	C15656T	Ts	T739I	Non-syn	0	neutral (-69)	0	0	1
nsp12 A3	A15657G	Ts	T739	Syn			2	1	3
nsp12 A3	A15659G	Ts	D740G	Non-syn	1	neutral (-27)	10	10	9
nsp12 A3	T15661C	Ts	F741L	Non-syn	2	neutral (-54)	6	1	5
nsp12 A3	T15662C	Ts	F741S	Non-syn	-3	effect (9)	1	0	0
nsp12 A3	T15665C	Ts	V742A	Non-syn	0	neutral (-13)	1	0	0
nsp12 A3	A15667G	Ts	N743D	Non-syn	2	neutral (-85)	10	10	10
nsp12 A4	T15669C	Ts	N743	Syn			9	9	10
nsp12 A3	A15671G	Ts	E744G	Non-syn	0	neutral (-33)	9	8	10
nsp12 A3/A4	T15673C	Ts	F745L	Non-syn	2	effect (23)	10	10	10
nsp12 A3	A15681G	Ts	A747	Syn			6	7	6
nsp12 A4	T15685C	Ts	L749	Syn			3	1	1
nsp12 A4	T15686C	Ts	L749S	Non-syn	-3	effect (50)	1	0	0
nsp12 A4	T15690C	Ts	R750	Syn			2	0	0
nsp12 A3	A15692G	Ts	K751R	Non-syn	3	effect (39)	3	9	5
nsp12 A4	T15696C	Ts	H752	Syn			6	5	5
nsp12 A4	T15698C	Ts	F753S	Non-syn	-3	effect (60)	0	0	1
nsp12 A4	T15700C	Ts	S754P	Non-syn	1	effect (6)	10	10	10
nsp12 A4	T15704C	Ts	M755T	Non-syn	-1	effect (73)	2	1	0

nsp12 A4	A15706G	Ts	M756V	Non-syn	2	neutral (-15)	1	0	1
nsp12 A4	T15707C	Ts	M756T	Non-syn	-1	effect (68)	4	2	3
nsp12 A4	A15709G	Ts	I757V	Non-syn	4	neutral (-29)	5	1	3
nsp12 A4	A15711G	Ts	I757M	Non-syn	2	effect (10)	1	0	0
nsp12 A4	C15712T	Ts	L758F	Non-syn	2	neutral (-43)	1	0	0
nsp12 A4	T15713C	Ts	L758P	Non-syn	-3	effect (46)	10	10	10
nsp12 A4	T15715C	Ts	S759P	Non-syn	1	effect (35)	9	10	10
nsp12 A4	T15717C	Ts	S759	Syn			6	3	4
nsp12 A4	T15717A	Tv	S759	Syn			1	0	0
nsp12 A4	A15719G[h]	Ts	D760G	Non-syn	1	effect (55)	7	3	8
nsp12 A4	G15721A[h]	Ts	D761N	Non-syn	2	effect (11)	1	0	0
nsp12 A4	T15723C	Ts	D761	Syn			1	1	1
nsp12 A4	T15726C	Ts	A762	Syn			10	10	10
nsp12 A4	T15728C	Ts	V763A	Non-syn	0	effect (10)	10	10	10
nsp12 A4	T15729C	Ts	V763	Syn			7	7	7
nsp12 A4	T15731C	Ts	V764A	Non-syn	0	neutral (-50)	10	10	10
nsp12 A4	T15733C	Ts	C765R	Non-syn	-4	effect (76)	10	10	10
nsp12 A4	T15735C	Ts	C765	Syn			10	10	10
nsp12 A4	T15736C	Ts	F766L	Non-syn	2	neutral (-59)	1	0	0
nsp12 A4	T15737C	Ts	F766S	Non-syn	-3	effect (37)	3	0	1
nsp12 A4	C15738T	Ts	F766	Syn			1	0	0
nsp12 A4	T15741C	Ts	N767	Syn			0	0	1
nsp12 A4	T15747C	Ts	T769	Syn			3	1	0
nsp12 A4	T15750C	Ts	Y770	Syn			5	6	7
nsp12 A4	A15753G	Ts	A771	Syn			1	0	1
nsp12 A4	T15754C	Ts	S772P	Non-syn	1	neutral (-88)	10	10	10
nsp12 A4	T15756C	Ts	S772	Syn			7	1	9
nsp12 A4	T15762C	Ts	G774	Syn			10	10	10
nsp12 A4	T15764C	Ts	L775P	Non-syn	-3	effect (29)	10	9	8
nsp12 A4	T15767C	Ts	V776A	Non-syn	0	neutral (-41)	8	8	7
nsp12 A4	T15771C	Ts	A777	Syn			10	9	8
nsp12 A4	A15772G	Ts	S778G	Non-syn	1	neutral (-28)	1	0	1
nsp12 A4	A15779G	Ts	K780R	Non-syn	3	neutral (-15)	1	0	0
nsp12 A4	A15782G	Ts	N781S	Non-syn	1	neutral (-58)	6	2	4
nsp12 A4	T15784C	Ts	F782L	Non-syn	2	effect (57)	9	9	9
nsp12 A4	A15788G	Ts	K783R	Non-syn	3	neutral (-85)	3	1	3
nsp12 A4	T15790C	Ts	S784P	Non-syn	1	neutral (-66)	9	10	8
nsp12 A4	A15792G	Ts	S784	Syn			2	1	0
nsp12 A4	T15794C	Ts	V785A	Non-syn	0	neutral (-73)	10	10	10
nsp12 A4	T15795C	Ts	V785	Syn			7	6	7
nsp12 A4	T15797C	Ts	L786P	Non-syn	-3	effect (67)	2	0	2

nsp12 A4	T15802C	Ts	Y788H	Non-syn	0	effect (72)	1	0	0
nsp12 A4	T15804C	Ts	Y788	Syn			5	1	1
nsp12 A4	A15808G	Ts	N790D	Non-syn	2	neutral (-3)	1	0	0
nsp12 A4	A15809G	Ts	N790S	Non-syn	1	neutral (-19)	5	3	8
nsp12 A4	T15815C	Ts	V792A	Non-syn	0	effect (48)	4	1	2
nsp12 A4	T15816C	Ts	V792	Syn			4	0	1
nsp12 A4	T15821C	Ts	M794T	Non-syn	-1	neutral (-22)	0	3	2
nsp12 A4	G15822T	Tv	M794I	Non-syn	2	neutral (0)	0	0	1
nsp12 A4	T15823C	Ts	S795P	Non-syn	1	neutral (-19)	10	10	10
nsp12 A4	T15825C	Ts	S795	Syn			0	0	1
nsp12 A4	A15827G	Ts	E796G	Non-syn	0	effect (29)	8	4	6
nsp12 A4	A15828G	Ts	E796	Syn			10	9	9
nsp12 A4	A15831G	Ts	A797	Syn			2	1	1
nsp12 A4	A15833G	Ts	K798R	Non-syn	3	neutral (-7)	9	9	7
nsp12 A4	A15834G	Ts	K798	Syn			4	4	5
nsp12 A4	T15837C	Ts	C799	Syn			3	0	2
nsp12 A4	T15838C	Ts	W800R	Non-syn	2	effect (57)	1	0	1
nsp12 A4	G15839A	Ts	W800*	Non-syn		effect (41)	1	0	0
nsp12 A4	A15841G	Ts	T801A	Non-syn	1	neutral (-45)	10	10	10
nsp12 A4	T15843C	Ts	T801	Syn			3	1	4
nsp12 A4	G15844A	Ts	E802K	Non-syn	0	effect (27)	1	0	0
nsp12 A4	A15845G	Ts	E802G	Non-syn	0	effect (22)	9	10	10
nsp12 A4	A15847G	Ts	T803A	Non-syn	1	neutral (-81)	10	10	10
nsp12 A4	T15849C	Ts	T803	Syn			2	2	2
nsp12 A4	A15851G	Ts	D804G	Non-syn	1	effect (21)	10	10	10
nsp12 A4	T15854C	Ts	L805P	Non-syn	-3	effect (59)	7	7	8
nsp12 A4	A15860G	Ts	K807R	Non-syn	3	neutral (-91)	2	0	0
nsp12 A4	A15861G	Ts	K807	Syn			10	10	10
nsp12 A4	A15864G	Ts	G808	Syn			9	9	8
nsp12 A4	T15867C	Ts	P809	Syn			10	10	10
nsp12 A4	C15868T	Ts	H810Y	Non-syn	0	effect (52)	0	0	1
nsp12 A4	A15869G	Ts	H810R	Non-syn	2	effect (62)	2	0	0
nsp12 A4	T15870C	Ts	H810	Syn			6	2	3
nsp12 A4	T15880C	Ts	S814P	Non-syn	1	effect (27)	10	10	10
nsp12 A4	T15882C	Ts	S814	Syn			10	10	10
nsp12 A4	A15884G	Ts	Q815R	Non-syn	1	effect (34)	2	0	0
nsp12 A4	A15885G	Ts	Q815	Syn			8	8	7
nsp12 A4	A15887G	Ts	H816R	Non-syn	2	neutral (-28)	4	5	5
nsp12 A4	A15891G	Ts	T817	Syn			8	5	5
nsp12 A4	A15892G	Ts	M818V	Non-syn	2	neutral (-85)	10	10	10
nsp12 A4	T15896C	Ts	L819P	Non-syn	-3	effect (62)	1	0	0

nsp12 A4	A15897G	Ts	L819	Syn			4	0	1
nsp12 A4	T15899C	Ts	V820A	Non-syn	0	neutral (-54)	1	0	1
nsp12 A4	A15903G	Ts	K821	Syn			5	3	4
nsp12 A4	A15905G	Ts	Q822R	Non-syn	1	neutral (-81)	8	4	6
nsp12 A4	G15906T[i]	Tv	Q822H	Non-syn	3	neutral (-85)	0	0	1
nsp12 A4	T15909C	Ts	G823	Syn			3	2	4
nsp12 A4	G15910A	Ts	D824N	Non-syn	2	neutral (-12)	1	0	0
nsp12 A4	A15911G	Ts	D824G	Non-syn	1	neutral (-50)	7	3	5
nsp12 A4	A15914G	Ts	D825G	Non-syn	1	neutral (-69)	1	0	0
nsp12 A4	T15915C	Ts	D825	Syn			1	0	0
nsp12 A4	T15920C	Ts	V827A	Non-syn	0	neutral (-53)	10	9	10
nsp12 A4	T15922C	Ts	Y828H	Non-syn	0	effect (58)	1	0	0
nsp12 A4	T15926C	Ts	L829P	Non-syn	-3	effect (80)	10	10	10
nsp12 A4	T15930C	Ts	P830	Syn			5	3	5
nsp12 A4	A15936G	Ts	P832	Syn			4	1	2
nsp12 A4	A15938G[h]	Ts	D833G	Non-syn	1	effect (67)	8	7	9
nsp12 A4	T15939C	Ts	D833	Syn			9	8	8
nsp12 A4	A15942G	Ts	P834	Syn			2	1	0
nsp12 A4	T15943C	Ts	S835P	Non-syn	1	effect (10)	2	0	2
nsp12 A4	C15944T	Ts	S835L	Non-syn	-3	neutral (0)	2	0	0
nsp12 A4	A15945G	Ts	S835	Syn			2	2	1
nsp12 A4	A15946G	Ts	R836G	Non-syn	-3	effect (88)	4	1	1
nsp12 A4	A15948G	Ts	R836	Syn			8	8	10
nsp12 A4	A15949G	Ts	I837V	Non-syn	4	neutral (-63)	8	9	5
nsp12 A4	A15954G	Ts	L838	Syn			4	3	3
nsp12 A4	T15964C[h]	Ts	C842R	Non-syn	-4	effect (59)	1	0	0
nsp12 A4	T15966C	Ts	C842	Syn			6	6	5
nsp12 A4	T15968C	Ts	F843S	Non-syn	-3	neutral (-24)	1	1	0
nsp12 A4	T15971C	Ts	V844A	Non-syn	0	effect (10)	1	0	0
nsp12 A4	A15972G	Ts	V844	Syn			0	1	1
nsp12 A4	A15977G	Ts	D846G	Non-syn	1	effect (57)	7	7	5
nsp12 A4	A15979G	Ts	I847V	Non-syn	4	neutral (-93)	1	0	0
nsp12 A4	T15980C	Ts	I847T	Non-syn	0	neutral (-67)	1	1	0
nsp12 A4	A15987G	Ts	K849	Syn			7	9	6
nsp12 A4	A15988G	Ts	T850A	Non-syn	1	neutral (-69)	4	6	2
nsp12 A4	A15992G[h]	Ts	D851G	Non-syn	1	effect (60)	10	10	10
nsp12 A4	T15996C	Ts	G852	Syn			1	0	0
nsp12 A4	A15997G	Ts	T853A	Non-syn	1	neutral (-29)	3	1	1
nsp12 A4	A15999G	Ts	T853	Syn			10	9	10
nsp12 A4	T16001C	Ts	L854P	Non-syn	-3	neutral (-43)	1	0	1
nsp12 A4	A16006G	Ts	I856V	Non-syn	4	neutral (-86)	10	10	10

nsp12 A4	A16010G	Ts	E857G	Non-syn	0	effect (40)	9	8	9
nsp12 A4	A16011G	Ts	E857	Syn			0	0	1
nsp12 A4	T16015C	Ts	F859L	Non-syn	2	neutral (-74)	7	8	9
nsp12 A4	T16016C	Ts	F859S	Non-syn	-3	effect (1)	3	1	2
nsp12 A4	T16019C	Ts	V860A	Non-syn	0	neutral (-37)	3	1	1
nsp12 A4	T16021C	Ts	S861P	Non-syn	1	effect (73)	6	4	4
nsp12 A4	T16023C	Ts	S861	Syn			4	0	2
nsp12 A4	A16026G	Ts	L862	Syn			1	1	0
nsp12 A4	C16028T	Ts	A863V	Non-syn	0	effect (11)	1	0	0
nsp12 A4	A16030G	Ts	I864V	Non-syn	4	neutral (-51)	2	1	0
nsp12 A4	A16034G	Ts	D865G	Non-syn	1	effect (81)	8	7	8
nsp12 A4	T16038C	Ts	A866	Syn			1	0	0
nsp12 A4	A16044G	Ts	P868	Syn			2	1	2
nsp12 A4	T16047C	Ts	L869	Syn			1	0	0
nsp12 A4	C16049T	Ts	T870I	Non-syn	0	neutral (-81)	0	0	1
nsp12 A4	A16052G	Ts	K871R	Non-syn	3	neutral (-86)	1	0	0
nsp12 A4	A16053G	Ts	K871	Syn			8	9	8
spike A5	T22873C	Ts	N437	Syn			7	10	8
spike A5	T22874C	Ts	S438P	Non-syn	1	neutral (-50)	8	10	9
spike A5	T22876C	Ts	S438	Syn			8	10	9
spike A5	T22882C	Ts	N440	Syn			7	8	8
spike A5	T22884C	Ts	L441P	Non-syn	-3	effect (53)	4	7	4
spike A5	T22885C	Ts	L441	Syn			0	1	0
spike A5	T22888C	Ts	D442	Syn			8	10	8
spike A5	T22889C	Ts	S443P	Non-syn	1	neutral (-9)	4	2	3
spike A5	A22893G	Ts	K444R	Non-syn	3	neutral (-58)	1	0	0
spike A5	T22896C	Ts	V445A	Non-syn	0	neutral (-50)	6	7	3
spike A5	T22897C	Ts	V445	Syn			0	2	0
spike A5	T22900C	Ts	G446	Syn			5	2	2
spike A5	T22917C	Ts	L452P	Non-syn	-3	neutral (-16)	8	10	8
spike A5	T22928C	Ts	F456L	Non-syn	2	neutral (-67)	3	3	1
spike A5	T22929C	Ts	F456S	Non-syn	-3	neutral (-32)	3	2	1
spike A5	A22934G	Ts	K458E	Non-syn	0	neutral (-31)	0	2	2
spike A5	A22935G	Ts	K458R	Non-syn	3	neutral (-76)	8	10	9
spike A5	T22937C	Ts	S459P	Non-syn	1	neutral (-28)	8	10	8
spike A5	T22939C	Ts	S459	Syn			0	1	1
spike A5	T22942C	Ts	N460	Syn			8	10	9
spike A5	T22944C	Ts	L461P	Non-syn	-3	effect (76)	8	10	9
spike A5	A22946G	Ts	K462E	Non-syn	0	effect (62)	0	1	0
spike A5	A22948G	Ts	K462	Syn			6	6	6
spike A5	T22951C	Ts	P463	Syn			8	10	9

spike A5	T22952C	Ts	F464L	Non-syn	2	neutral (-59)	1	0	0
spike A5	A22956G	Ts	E465G	Non-syn	0	neutral (-36)	8	10	9
spike A5	A22958G	Ts	R466G	Non-syn	-3	effect (11)	8	10	9
spike A5	A22960G	Ts	R466	Syn			8	10	9
spike A5	A22962G	Ts	D467G	Non-syn	1	neutral (-14)	8	10	9
spike A5	A22964G	Ts	I468V	Non-syn	4	neutral (-88)	0	1	0
spike A5	T22965C	Ts	I468T	Non-syn	0	neutral (-23)	1	0	0
spike A5	T22967C	Ts	S469P	Non-syn	1	neutral (-48)	1	1	0
spike A5	A22976G	Ts	I472V	Non-syn	4	neutral (-95)	4	5	0
spike A5	T22977C	Ts	I472T	Non-syn	0	neutral (-70)	3	2	0
spike A5	T22981C	Ts	Y473	Syn			0	1	0
spike A5	A22983G	Ts	Q474R	Non-syn	1	neutral (-60)	3	5	6
spike A5	C22986T	Ts	A475V	Non-syn	0	neutral (-88)	1	0	0
spike A5	T22990C	Ts	G476	Syn			4	4	1
spike A5	A22991G	Ts	S477G	Non-syn	1	neutral (-60)	0	0	1
spike A5	A22994G	Ts	T478A	Non-syn	1	neutral (-73)	5	5	3
spike A5	A22996G	Ts	T478	Syn			6	5	5
spike A5	C22997T	Ts	P479S	Non-syn	1	neutral (-16)	1	0	0
spike A5	T22999C	Ts	P479	Syn			7	9	8
spike A5	A23004G	Ts	N481S	Non-syn	1	neutral (-90)	2	1	1
spike A5	T23005C	Ts	N481	Syn			2	2	0
spike A5	T23008C	Ts	G482	Syn			7	6	5
spike A5	T23010C	Ts	V483A	Non-syn	0	neutral (-69)	8	8	9
spike A5	T23011C	Ts	V483	Syn			1	1	0
spike A5	A23014G	Ts	E484	Syn			8	7	8
spike A5	T23017C	Ts	G485	Syn			8	10	9
spike A5	T23026C	Ts	C488	Syn			8	9	8
spike A5	T23030C	Ts	F490L	Non-syn	2	neutral (-59)	8	10	9
spike A5	T23032C	Ts	F490	Syn			7	6	8
spike A5	T23035C	Ts	P491	Syn			7	6	7
spike A5	T23036C	Ts	L492	Syn			4	2	3
spike A5	A23041G	Ts	Q493	Syn			2	2	1
spike A5	T23042C[i]	Ts	S494P	Non-syn	1	neutral (-25)	3	3	0
spike A5	A23046G	Ts	Y495C	Non-syn	0	effect (4)	0	0	1
spike A5	T23047C	Ts	Y495	Syn			2	1	0
spike A5	T23050C	Ts	G496	Syn			7	10	9
spike A5	T23051C	Ts	F497L	Non-syn	2	neutral (-79)	3	3	0
spike A5	T23052C	Ts	F497S	Non-syn	-3	effect (4)	1	1	0
spike A5	A23055G	Ts	Q498R	Non-syn	1	neutral (-52)	1	1	0
spike A5	A23056G	Ts	Q498	Syn			8	5	6
spike A5	C23061T	Ts	T500I	Non-syn	0	neutral (-6)	1	0	0

spike A5	T23065C	Ts	N501	Syn			1	0	0
spike A5	T23068C	Ts	G502	Syn			6	5	7
spike A5	T23070C	Ts	V503A	Non-syn	0	neutral (-55)	7	9	7
spike A5	T23074C	Ts	G504	Syn			5	6	6
spike A5	A23076G	Ts	Y505C	Non-syn	0	effect (6)	0	1	0
spike A5	A23079G	Ts	Q506R	Non-syn	1	effect (21)	3	2	1
spike A5	A23080G	Ts	Q506	Syn			4	2	2
spike A5	A23087G	Ts	R509G	Non-syn	-3	neutral (-32)	5	7	5
spike A5	A23089G	Ts	R509	Syn			8	10	9
spike A5	A23095G	Ts	V511	Syn			1	0	0
spike A5	T23100C	Ts	L513P	Non-syn	-3	effect (73)	8	10	9
spike A5	T23102C	Ts	S514P	Non-syn	1	effect (9)	5	6	8
spike A5	T23104C	Ts	S514	Syn			8	10	9
spike A5	A23109G	Ts	E516G	Non-syn	0	neutral (-49)	5	2	1
spike A5	A23110G	Ts	E516	Syn			6	4	6
spike A5	T23112C	Ts	L517P	Non-syn	-3	effect (11)	8	10	9
spike A5	A23116G	Ts	L518	Syn			4	1	0
spike A5	A23118G	Ts	H519R	Non-syn	2	neutral (-48)	5	3	4
spike A5	A23122G	Ts	A520	Syn			8	10	9
spike A5	A23125G	Ts	P521	Syn			4	2	3
spike A5	C23127T[i]	Ts	A522V	Non-syn	0	neutral (-71)	0	0	1
spike A5	A23129G	Ts	T523A	Non-syn	1	neutral (-45)	8	8	8
spike A5	T23131C	Ts	T523	Syn			0	1	0
spike A5	T23133C	Ts	V524A	Non-syn	0	effect (41)	5	7	8
spike A5	T23134C	Ts	V524	Syn			1	0	0
spike A5	T23137C	Ts	C525	Syn			1	0	2
spike A5	A23140G	Ts	G526	Syn			8	10	9
spike A5	A23148G	Ts	K529R	Non-syn	3	neutral (-71)	2	2	2
spike A5	T23150C	Ts	S530P	Non-syn	1	neutral (-36)	8	10	9
spike A5	T23152C	Ts	S530	Syn			2	0	1
spike A5	A23153G	Ts	T531A	Non-syn	1	neutral (-63)	0	0	1
spike A5	T23159C	Ts	L533	Syn			0	1	0
spike A5	T23163C	Ts	V534A	Non-syn	0	neutral (-4)	4	1	4
spike A5	A23168G	Ts	N536D	Non-syn	2	neutral (-50)	5	5	1
spike A5	A23169G	Ts	N536S	Non-syn	1	neutral (-50)	8	10	9
spike A5	A23172G	Ts	K537R	Non-syn	3	neutral (-35)	2	1	0
spike A5	A23173G	Ts	K537	Syn			2	2	0
spike A5	T23176C	Ts	C538	Syn			7	7	9
spike A5	T23178C	Ts	V539A	Non-syn	0	effect (3)	8	10	9
spike A5	A23180G	Ts	N540D	Non-syn	2	neutral (-68)	0	1	0
spike A5	T23182C	Ts	N540	Syn			8	10	8

spike A5	T23184C	Ts	F541S	Non-syn	-3	effect (87)	0	1	0
spike A5	A23187G	Ts	N542S	Non-syn	1	neutral (-12)	2	2	0
spike A5	C23188T	Ts	N542	Syn			0	1	0
spike A5	T23189C	Ts	F543L	Non-syn	2	neutral (-61)	3	4	3
spike A5	A23193G	Ts	N544S	Non-syn	1	neutral (-67)	2	2	0
spike A5	T23197C	Ts	G545	Syn			1	0	0
spike A5	A23201G	Ts	T547A	Non-syn	1	effect (12)	5	8	7
spike A5	C23202T	Ts	T547I	Non-syn	0	effect (72)	0	0	1
spike A5	A23203G	Ts	T547	Syn			8	10	9
spike A5	A23207G	Ts	T549A	Non-syn	1	neutral (-33)	8	9	8
spike A5	A23209G	Ts	T549	Syn			8	7	7
spike A5	T23212C	Ts	G550	Syn			1	1	0
spike A5	T23214C	Ts	V551A	Non-syn	0	effect (34)	8	9	9
spike A5	T23215C	Ts	V551	Syn			8	7	7
spike A5	T23217C	Ts	L552P	Non-syn	-3	effect (89)	2	0	1
spike A5	A23223G	Ts	E554G	Non-syn	0	effect (12)	8	10	9
spike A5	T23225C	Ts	S555P	Non-syn	1	effect (31)	7	9	7
spike A5	A23229G	Ts	N556S	Non-syn	1	neutral (-56)	7	10	7
spike A5	A23233G	Ts	K557	Syn			2	1	0
spike A5	A23235G	Ts	K558R	Non-syn	3	neutral (-86)	0	1	0
spike A5	T23241C	Ts	L560P	Non-syn	-3	neutral (-11)	5	5	2
spike A5	T23245C	Ts	P561	Syn			8	10	8
spike A5	T23246C	Ts	F562L	Non-syn	2	neutral (-12)	4	3	4
spike A5	T23247C	Ts	F562S	Non-syn	-3	effect (21)	3	4	4
spike A5	A23250G	Ts	Q563R	Non-syn	1	neutral (-21)	3	2	1
spike A5	A23251G	Ts	Q563	Syn			8	8	6
spike A5	A23253G	Ts	Q564R	Non-syn	1	effect (15)	0	1	0
spike A5/A6	A23261G	Ts	R567G	Non-syn	-3	effect (47)	9	10	8
spike A5/A6	A23263G	Ts	R567	Syn			10	10	10
spike A5/A6	A23265G	Ts	D568G	Non-syn	1	neutral (-5)	9	10	9
spike A5	A23267G	Ts	I569V	Non-syn	4	neutral (-76)	3	2	0
spike A6	T23268C	Ts	I569T	Non-syn	0	neutral (-49)	1	0	0
spike A6	C23271T	Ts	A570V	Non-syn	0	neutral (-88)	0	1	0
spike A6	T23272C	Ts	A570	Syn			9	9	9
spike A6	G23273A	Ts	D571N	Non-syn	2	neutral (-46)	1	0	0
spike A6	A23274G	Ts	D571G	Non-syn	1	neutral (-61)	3	0	0
spike A6	A23276G	Ts	T572A	Non-syn	1	neutral (-66)	6	1	4
spike A6	T23278C	Ts	T572	Syn			1	0	0
spike A6	C23280T	Ts	T573I	Non-syn	0	neutral (-54)	1	0	0
spike A6	T23281C	Ts	T573	Syn			4	1	4
spike A6	A23283G	Ts	D574G	Non-syn	1	neutral (-39)	2	0	1

spike A6	T23284C	Ts	D574	Syn			7	6	6
spike A6	C23286T	Ts	A575V	Non-syn	0	neutral (-71)	1	0	0
spike A6	T23287C	Ts	A575	Syn			9	8	10
spike A6	T23289C	Ts	V576A	Non-syn	0	neutral (-35)	10	10	10
spike A6	T23293C	Ts	R577	Syn			10	8	8
spike A6	A23295G	Ts	D578G	Non-syn	1	effect (42)	4	1	4
spike A6	T23296C	Ts	D578	Syn			10	7	8
spike A6	A23299G	Ts	P579	Syn			1	0	0
spike A6	A23301G	Ts	Q580R	Non-syn	1	neutral (-38)	4	5	4
spike A6	G23302T	Tv	Q580H	Non-syn	3	neutral (-42)	0	1	0
spike A6	A23303G	Ts	T581A	Non-syn	1	effect (42)	9	8	7
spike A6	A23305G	Ts	T581	Syn			3	1	0
spike A6	T23307C	Ts	L582P	Non-syn	-3	effect (28)	7	8	8
spike A6	T23308C	Ts	L582	Syn			3	1	3
spike A6	A23310G	Ts	E583G	Non-syn	0	neutral (-12)	9	6	9
spike A6	G23311T	Tv	E583D	Non-syn	3	neutral (-59)	0	0	1
spike A6	A23312G	Ts	I584V	Non-syn	4	neutral (-95)	1	2	2
spike A6	T23313C	Ts	I584T	Non-syn	0	neutral (-68)	3	0	1
spike A6	T23314C	Ts	I584	Syn			2	0	1
spike A6	T23317C	Ts	L585	Syn			3	0	2
spike A6	A23319G	Ts	D586G	Non-syn	1	effect (5)	9	6	8
spike A6	A23321G	Ts	I587V	Non-syn	4	neutral (-74)	1	0	0
spike A6	T23322C	Ts	I587T	Non-syn	0	effect (55)	3	0	3
spike A6	A23326G	Ts	T588	Syn			9	8	7
spike A6	T23330C	Ts	C590R	Non-syn	-4	effect (93)	8	8	8
spike A6	T23332C	Ts	C590	Syn			10	10	10
spike A6	T23333C	Ts	S591P	Non-syn	1	neutral (-25)	9	7	8
spike A6	T23335C	Ts	S591	Syn			9	8	8
spike A6	T23337C	Ts	F592S	Non-syn	-3	neutral (-68)	3	0	0
spike A6	T23338C	Ts	F592	Syn			1	0	0
spike A6	T23341C	Ts	G593	Syn			6	2	5
spike A6	T23344C	Ts	G594	Syn			10	10	8
spike A6	T23346C	Ts	V595A	Non-syn	0	effect (10)	10	10	10
spike A6	A23348G	Ts	S596G	Non-syn	1	effect (49)	0	1	2
spike A6	T23350C	Ts	S596	Syn			10	10	10
spike A6	T23352C	Ts	V597A	Non-syn	0	neutral (-50)	10	10	10
spike A6	A23357G	Ts	T599A	Non-syn	1	neutral (-21)	3	1	3
spike A6	A23359G	Ts	T599	Syn			7	5	1
spike A6	A23362G	Ts	P600	Syn			5	0	0
spike A6	A23365G	Ts	G601	Syn			0	0	1
spike A6	A23366G	Ts	T602A	Non-syn	1	neutral (-65)	10	10	10

spike A6	A23370G	Ts	N603S	Non-syn	1	neutral (-20)	1	1	1
spike A6	T23374C	Ts	T604	Syn			10	10	10
spike A6	A23379G	Ts	N606S	Non-syn	1	neutral (-68)	4	0	1
spike A6	C23380T[i]	Ts	N606	Syn			1	2	0
spike A6	T23385C	Ts	V608A	Non-syn	0	neutral (-59)	9	10	9
spike A6	T23386C	Ts	V608	Syn			1	1	0
spike A6	T23389C	Ts	A609	Syn			8	8	6
spike A6	T23391C	Ts	V610A	Non-syn	0	neutral (-7)	10	10	10
spike A6	T23392C	Ts	V610	Syn			6	5	8
spike A6	T23394C	Ts	L611P	Non-syn	-3	effect (85)	10	10	10
spike A6	T23398C	Ts	Y612	Syn			2	0	0
spike A6	C23399T	Ts	Q613*	Non-syn		neutral (-57)	1	0	0
spike A6	A23400G	Ts	Q613R	Non-syn	1	neutral (-61)	9	5	7
spike A6	A23403G[i]	Ts	D614G	Non-syn	1	neutral (-52)	10	9	10
spike A6	A23403G[i]+ T23404C[j]	Ts	D614G	Non-syn	1	neutral (-52)	8	6	5
spike A6	T23406C	Ts	V615A	Non-syn	0	neutral (-30)	10	8	9
spike A6	A23409G	Ts	N616S	Non-syn	1	neutral (-85)	2	3	1
spike A6	T23411C	Ts	C617R	Non-syn	-4	effect (84)	7	5	7
spike A6	A23414G	Ts	T618A	Non-syn	1	neutral (-60)	10	10	9
spike A6	A23416G	Ts	T618	Syn			1	0	0
spike A6	A23419G	Ts	E619	Syn			10	10	10
spike A6	T23421C	Ts	V620A	Non-syn	0	neutral (-12)	10	10	10
spike A6	T23425C	Ts	P621	Syn			8	2	3
spike A6	G23426A	Ts	V622I	Non-syn	4	neutral (-56)	1	0	0
spike A6	T23427C	Ts	V622A	Non-syn	0	neutral (-50)	8	9	9
spike A6	T23428C	Ts	V622	Syn			7	6	5
spike A6	T23433C	Ts	I624T	Non-syn	0	effect (8)	5	2	7
spike A6	T23434C	Ts	I624	Syn			5	4	4
spike A6	A23436G	Ts	H625R	Non-syn	2	neutral (-14)	2	0	1
spike A6	T23437C	Ts	H625	Syn			3	1	0
spike A6	A23440G	Ts	A626	Syn			10	10	10
spike A6	A23442G	Ts	D627G	Non-syn	1	neutral (-14)	1	0	1
spike A6	T23443C	Ts	D627	Syn			4	0	2
spike A6	A23445G	Ts	Q628R	Non-syn	1	neutral (-22)	3	0	1
spike A6	A23446G[h]	Ts	Q628G	Non-syn	-1	neutral (0)	1	1	2
spike A6	T23448C	Ts	L629P	Non-syn	-3	effect (17)	5	2	4
spike A6	T23452C	Ts	T630	Syn			10	10	10
spike A6	T23455C	Ts	P631	Syn			1	0	0
spike A6	T23459C	Ts	W633R	Non-syn	2	effect (75)	3	0	3
spike A6	T23464C	Ts	R634	Syn			5	1	3

spike A6	T23466C	Ts	V635A	Non-syn	0	neutral (-16)	10	9	8
spike A6	T23467C	Ts	V635	Syn			0	0	2
spike A6	T23470C	Ts	Y636	Syn			2	0	4
spike A6	T23471C	Ts	S637P	Non-syn	1	effect (44)	1	0	0
spike A6	A23474G	Ts	T638A	Non-syn	1	effect (17)	1	0	1
spike A6	C23475T	Ts	T638I	Non-syn	0	effect (66)	1	0	0
spike A6	A23476G[h]	Ts	T638M	Non-syn	-1	effect (60)	9	10	8
spike A6	T23479C	Ts	G639	Syn			10	10	10
spike A6	T23480C	Ts	S640P	Non-syn	1	neutral (-45)	8	5	8
spike A6	C23481T	Ts	S640F	Non-syn	-3	neutral (-14)	1	0	0
spike A6	T23482C	Ts	S640	Syn			1	0	1
spike A6	T23485C	Ts	N641	Syn			1	0	0
spike A6	T23487C	Ts	V642A	Non-syn	0	neutral (-53)	10	10	9
spike A6	T23489C	Ts	F643L	Non-syn	2	effect (3)	8	6	8
spike A6	A23493G	Ts	Q644R	Non-syn	1	effect (34)	2	0	0
spike A6	A23495G	Ts	T645A	Non-syn	1	effect (52)	10	10	9
spike A6	A23497G	Ts	T645	Syn			7	8	5
spike A6	T23500C	Ts	R646	Syn			0	0	1
spike A6	A23503G	Ts	A647	Syn			10	10	10
spike A6	T23507C[h]	Ts	C649R	Non-syn	-4	effect (91)	3	1	4
spike A6	T23509C	Ts	C649	Syn			2	0	2
spike A6	A23513G	Ts	I651V	Non-syn	4	neutral (-86)	1	0	0
spike A6	A23515G	Ts	I651M	Non-syn	2	neutral (0)	2	1	2
spike A6	G23517A	Ts	G652E	Non-syn	0	effect (78)	1	0	0
spike A6	G23519A	Ts	A653T	Non-syn	1	neutral (-75)	1	0	0
spike A6	C23520T	Ts	A653V	Non-syn	0	neutral (-91)	1	0	0
spike A6	T23521C	Ts	A653	Syn			6	6	4
spike A6	A23523G	Ts	E654G	Non-syn	0	effect (44)	1	0	1
spike A6	A23524G	Ts	E654	Syn			10	10	10
spike A6	A23526G	Ts	H655R	Non-syn	2	effect (21)	2	1	3
spike A6	T23527C	Ts	H655	Syn			3	0	3
spike A6	T23529C	Ts	V656A	Non-syn	0	neutral (-41)	10	9	10
spike A6	A23531G	Ts	N657D	Non-syn	2	neutral (-21)	9	9	9
spike A6	A23532G	Ts	N657S	Non-syn	1	neutral (-81)	3	0	3
spike A6	A23535G	Ts	N658S	Non-syn	1	neutral (-63)	3	1	4
spike A6	T23537C	Ts	S659P	Non-syn	1	effect (23)	5	4	5
spike A6	A23544G	Ts	E661G	Non-syn	0	effect (41)	9	5	9
spike A6	T23546C	Ts	C662R	Non-syn	-4	effect (92)	4	0	2
spike A6	T23548C	Ts	C662	Syn			8	4	7
spike A6	A23550G	Ts	D663G	Non-syn	1	neutral (-8)	10	10	10
spike A6	A23552G	Ts	I664V	Non-syn	4	neutral (-60)	2	0	3

spike A6	A23554G	Ts	I664M	Non-syn	2	neutral (-75)	1	0	1
spike A6	T23563C	Ts	G667	Syn			1	0	0
spike A6	A23566G	Ts	A668	Syn			10	9	10
spike A6	T23569C	Ts	G669	Syn			1	0	0
spike A6	A23572G	Ts	I670M	Non-syn	2	neutral (-45)	1	0	0
spike A6	T23581C	Ts	S673	Syn			1	0	1
spike A6	T23582C	Ts	Y674H	Non-syn	0	effect (26)	1	0	0
spike A6	T23584C	Ts	Y674	Syn			2	0	1
spike A6	A23586G	Ts	Q675R	Non-syn	1	neutral (-15)	7	2	4
spike A6	A23588G	Ts	T676A	Non-syn	1	neutral (-29)	10	10	10
spike A6	T23590C	Ts	T676	Syn			1	0	3
spike A6	A23592G	Ts	Q677R	Non-syn	1	neutral (-52)	3	1	4
spike A6	A23594G	Ts	T678A	Non-syn	1	neutral (-23)	10	10	10
spike A6	A23598G	Ts	N679S	Non-syn	1	neutral (-22)	1	0	1
spike A6	T23600C	Ts	S680P	Non-syn	1	effect (24)	1	0	2
spike A6	T23602C	Ts	S680	Syn			1	1	2
spike A6	T23605C	Ts	P681	Syn			1	0	0
spike A6	G23607A	Ts	R682Q	Non-syn	1	effect (24)	1	0	0
spike A6	C23609T	Ts	R683W	Non-syn	2	effect (81)	1	0	0
spike A6	A23614G	Ts	A684	Syn			6	0	4
spike A6	C23615T	Ts	R685C	Non-syn	-4	effect (26)	1	0	0
spike A6	T23620C	Ts	S686	Syn			1	0	0
spike A6	A23623G	Ts	V687	Syn			6	2	2
spike A6	T23626C	Ts	A688	Syn			1	0	0
spike A6	T23629C	Ts	S689	Syn			1	0	0
spike A6	A23631G	Ts	Q690R	Non-syn	1	neutral (-19)	1	0	0
spike A6	A23632G	Ts	Q690	Syn			1	0	0
spike A6	T23633C	Ts	S691P	Non-syn	1	neutral (-79)	2	0	0
spike A6	A23636G	Ts	I692V	Non-syn	4	neutral (-62)	2	2	3
spike A6	A23639G	Ts	I693V	Non-syn	4	neutral (-86)	1	0	0
spike A6	T23645C	Ts	Y695H	Non-syn	0	effect (57)	1	0	0

[a] Genomic region and amplicon. The genomic regions covered by each amplicon are depicted in Figure 1 of the main text.

[b] The SARS-CoV-2 genome residue numbering is according to the NCBI reference sequence (accession number NC_045512.2).

[c] Type of mutation: transition (Ts); transversion (Tv).

[d] Amino acid residues (single letter code) are numbered fron N- to the C- terminus of each protein.

[e] Type of substitution: synonymous (Syn), Non-synonymous (Non-syn).

[f] PAM250 (accepted point mutation 250) substitution matrix (Feng and Doolittle, Methods in Enzymology 266: 368-382, 1996). PAM250<0, lower acceptability than expected; PAM250=0, acceptability as expected; PAM250>0, acceptability higher than expected.

[g] SNAP2 (Screening for Non-Acceptable Polymorphism 2) prediction score values (Hecht et al, BMC Genomics 2015, 16 Suppl 8:S1). Values between -100 to 0 correspond to neutral substitution and values between 0 to 100 correspond to substitution with an effect.

[h] Mutations that have not been reported in database outbreak.info (enabled by GISAID) prior to March 24th, 2022.

[i] Divergence mutations: mutations detected at frequency of 90-100% counted relative to the Wuhan-Hu-1 NCBI reference sequence (NC_045512.2).

[j] Mutation T23404C is always present in the same haplotype as the divergence mutation A23403G.

Table S4. Deletions found in nsp12 (polymerase)- and spike-coding regions of SARS-CoV-2 isolated from infected patients.

Region[a]	Deleted nucleotides[b]	Affected amino acid positions[c]	Amino acid reference sequence[c,d]	Amino acid sequence detected[e]	Number of patients		
					Mild	Moderate	Exitus
nsp12 A1	14576 - 14627	379 – 396[e]	DPAMHAASGNLLLDKRTTCFSV	DPVQ*	1	0	0
nsp12 A1	14851 – 14853	471[e]	LLFVV	LLVV	2	2	0
nsp12 A1	14851 – 14856	471 – 472	LLFVVE	LLVE	5	4	4
nsp12 A1	14852 – 14853	471[e]	LLFVV	LLCS*	0	0	1
nsp12 A1	14852 – 14855	471 – 472	LLFVVE	LL*	1	0	0
nsp12 A1	14853 – 14855	471 – 472	LLFVVE	LLLVE	0	1	1
nsp12 A1	14853 - 14856	471 - 472	LLFVVE	LLLLKLLISTLIVTMVAV LMLTKSSSTT*	2	0	1
nsp12 A1	14856 – 14858	472 – 473	LFVVEV	LFVEV	4	7	6
nsp12 A1	14861 – 14866	474 – 476[e]	VVEVVDK	VVVDK	0	4	1
nsp12 A1	14901 – 14905	487 – 489	GGCINAN	GG*	1	0	0
nsp12 A1	14903 – 14904	488[e]	GCINA	GCKC*	0	1	0
nsp12 A1	14903 – 14905	488 – 489[e]	GCINAN	GCNAN	1	4	3
nsp12 A1	14903 – 14911	488 – 491	GCINANQV	GCNQV	1	0	0
nsp12 A1	14906 – 14912	489 – 491	CINANQV	CITKSSSTT*	1	0	0
nsp12 A2	15022 – 15040	528 – 534	ALFAYTKRNVI	ALMSSLL*	2	5	4
nsp12 A2	15023 – 15040	528 – 534	ALFAYTKRNVI	ALYVI	1	1	1
nsp12 A2	15203 – 15215	588 – 592[e]	TVVIGTSKF	TVANSMVVGTTC*	1	0	0
spike A6	23397 – 23398	612[e]	VLYQD	VLSGC*	1	0	0
spike A6	23433 – 23483	624 – 641[e]	VAIHADQLTPT WRVYSTGSNVF	VANVF	1	0	0
spike A6	23450 – 23456	630 – 632	QLTPTWR	QLLGVFILQVLMFFKHV QAV*	0	1	1
spike A6	23450 – 23459	630 – 633[e]	QLTPTWRV	QLGVFILQVLMFFKHVQ AV*	0	1	1
spike A6	23541 – 23584	660 – 674[e]	NSYECDIPIGAGICASYQT	NSSDSD*	0	0	1
spike A6	23542 – 23582	660 – 674[e]	NSYECDIPIGAGICASYQT	NS*	0	5	3
spike A6	23543 – 23584	661 – 674	SYECDIPIGAGICASYQT	NSYQT	0	0	1
spike A6	23551 – 23583	663 – 674[e]	ECDIPIGAGICASYQT	ECDQT	0	3	4

spike A6	23555 – 23570	665 – 670[e]	DIPIGAGICA	DIYALVIRLRLILLGGHVV*	1	3	3
spike A6	23555 – 23582	665 – 674	DIPIGAGICASYQT	DIIRLRLILLGGHVV*	9	10	10
spike A6	23561 – 23570	667 – 670	PIGAGICA	PIYALVIRLRLILLGGHVV*	0	1	1
spike A6	23561 – 23582	667 - 674	PIGAGICASYQT	PIIRLRLILLGGHVV*	5	10	9
spike A6	23562 – 23580	667 – 673	PIGAGICASYQ	PIVIRLRLILLGGHVV*	0	0	1
spike A6	23571 – 23582	670 – 674[e]	AGICASYQT	AGNQT	0	7	3
spike A6	23572 – 23573	670 – 671[e]	AGICAS	AGMR*	0	1	2
spike A6	23572 – 23584	670 – 674[e]	AGICASYQT	AGIRLRLILLGGHVV*	1	0	1

[a] Genomic region and amplicon. The genomic regions covered by each amplicon are depicted in Figure 1 of the main text.

[b] The SARS-CoV-2 genome residue numbering is according to the NCBI reference sequence (accession number NC 045512.2).

[c] Amino acid residues are numbered from N- to the C-terminus of each protein.

[d] Amino acids affected by the deletion are the ones underlined.

[e] Amino acid sequence detected in virus from infected patients. The asterisks indicate a stop codon. Absence of asterisk means that the open reading frame is maintained.

[f] Deletions that have not been reported in database outbreak.info (enabled by GISAID) prior to March 24th, 2022.

Table S5. Statistical analysis of the acceptability of the repertoire of amino acid substitutions identified in nsp12 (polymerase) and spike.

SNAP2 > 0	Cut-off 0.5%	Cut-off 0.1%	p-value	Significance[a]
**nsp12**	19	170	**0.863**	**n.s.**
**spike**	5	56	**1**	**n.s.**
**p-value**	**0.369**	**< 0.001**		
**Significance[a]**	**n.s.**	***		
**PAM250 < 0**	**Cut-off 0.5%**	**Cut-off 0.1%**	**p-value**	**Significance[a]**
**nsp12**	6	72	**0.468**	**n.s.**
**spike**	3	26	**1**	**n.s.**
**p-value**	**1**	**0.142**		
**Significance[a]**	**n.s.**	**n.s.**		

[a] Statistical significance of the differences is given (n.s. not significant; *** $p < 0.001$).

Figure S1

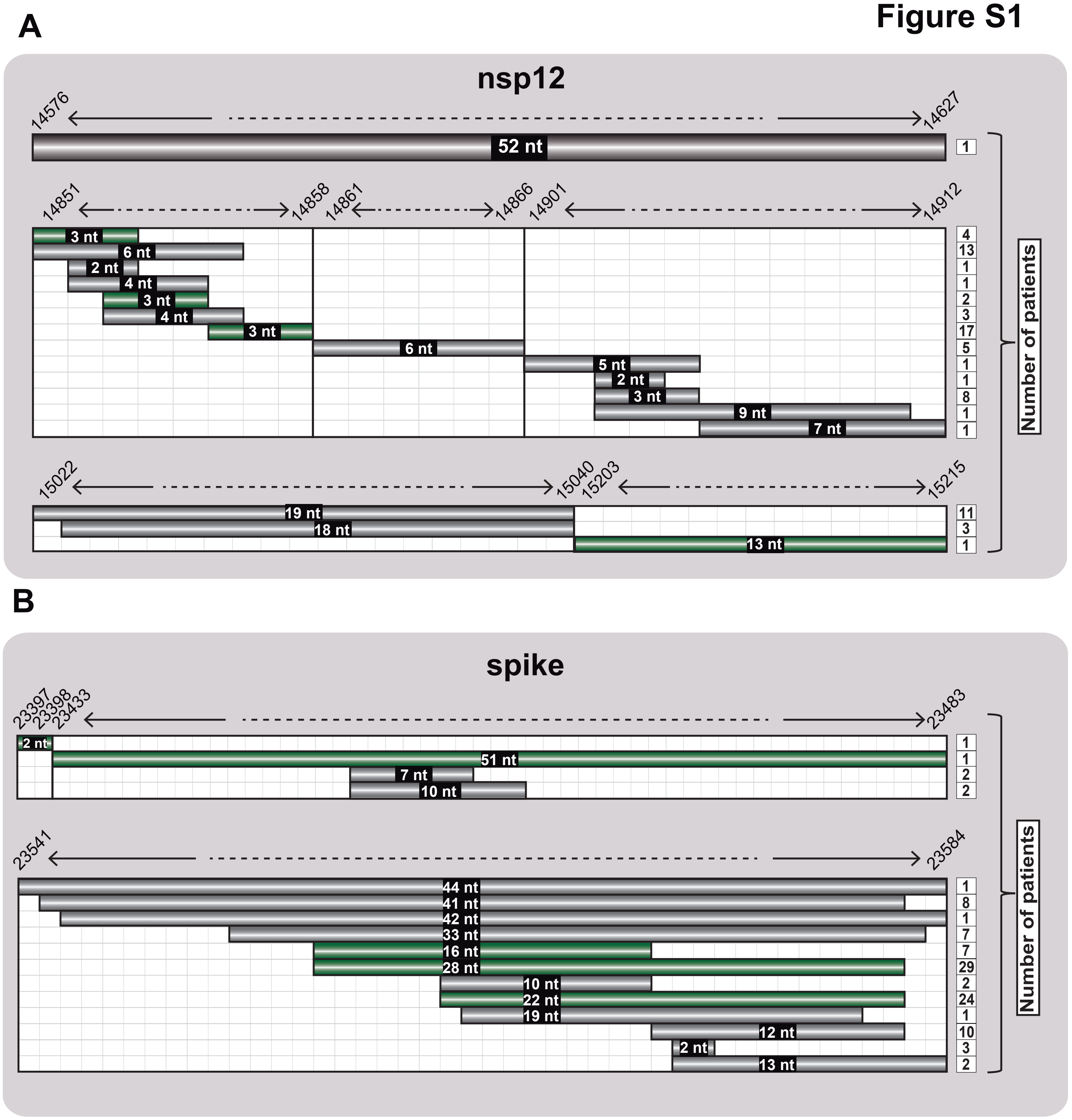

Figure S1. Deletion types in the nsp12 (polymerase)-(A) and the spike-(B) coding regions. Each deletion is indicated by a grey bar (deletions found only at 0.1% cut-off) or green (deletions found at 0.1% and 0.5% cut-off), with the number of deleted nucleotides indicated inside the black boxes. On the top, the numbering of the nucleotides covering each region is given (genome numbering is according to the reference genome NCBI accession number: NC_045512.2). The white boxes at the right indicate the total number of patients whose virus carries each deletion.

Figure S2

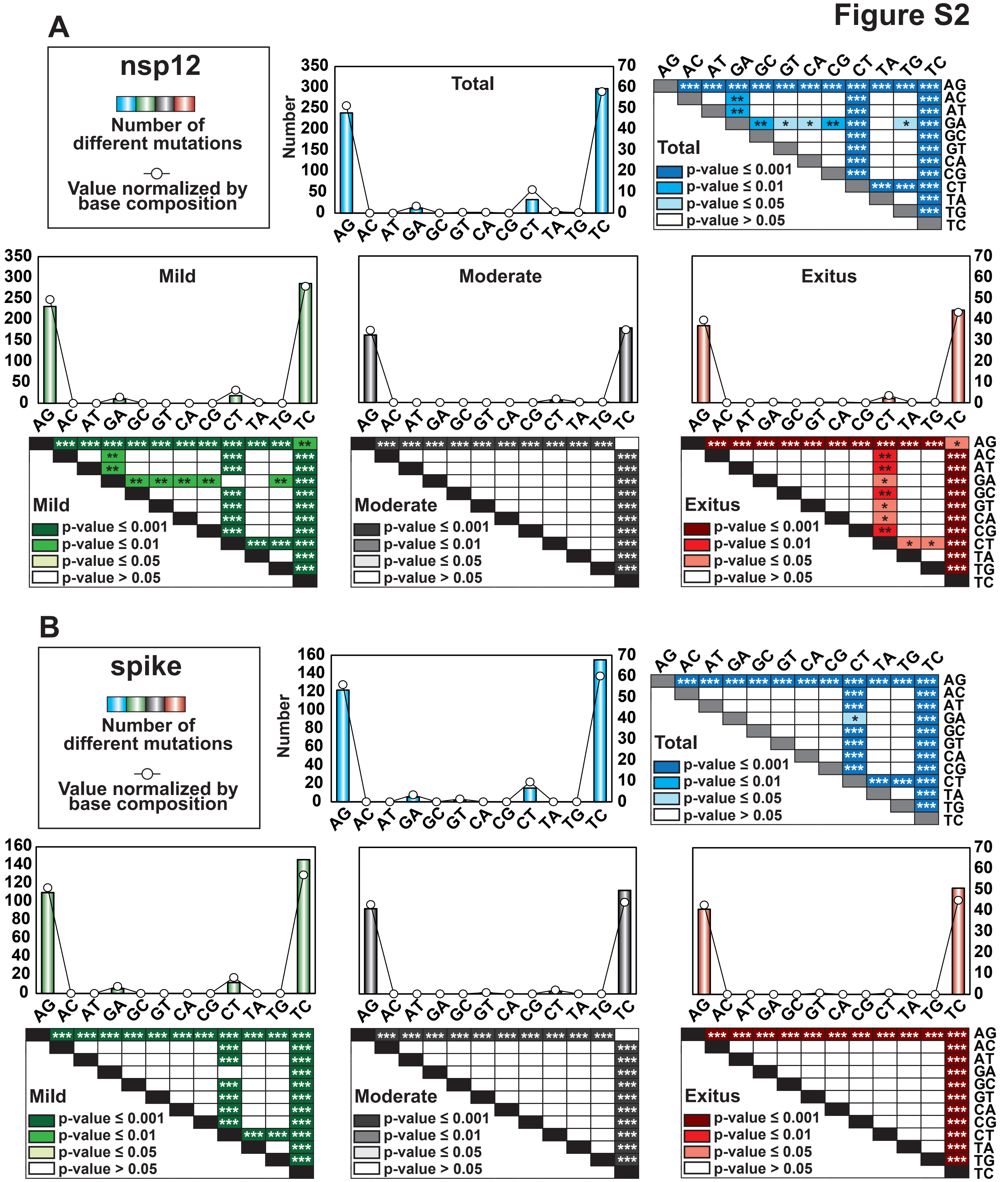

Figure S2. Mutation types in the mutant spectra of SARS-CoV-2. The distribution of the type of mutations and the statistical significance (proportion test) is given for the total number of different mutations (blue), and for the mutations divided according to the severity of COVID-19 (green for mild, grey for moderate, and red for exitus). Bars give total numbers, and the dots indicate the values normalized to the nucleotide composition of the genome in the sequenced regions. **(A), (B)** Data for the amplicons of the nsp12 (polymerase)- and spike- coding regions, respectively.

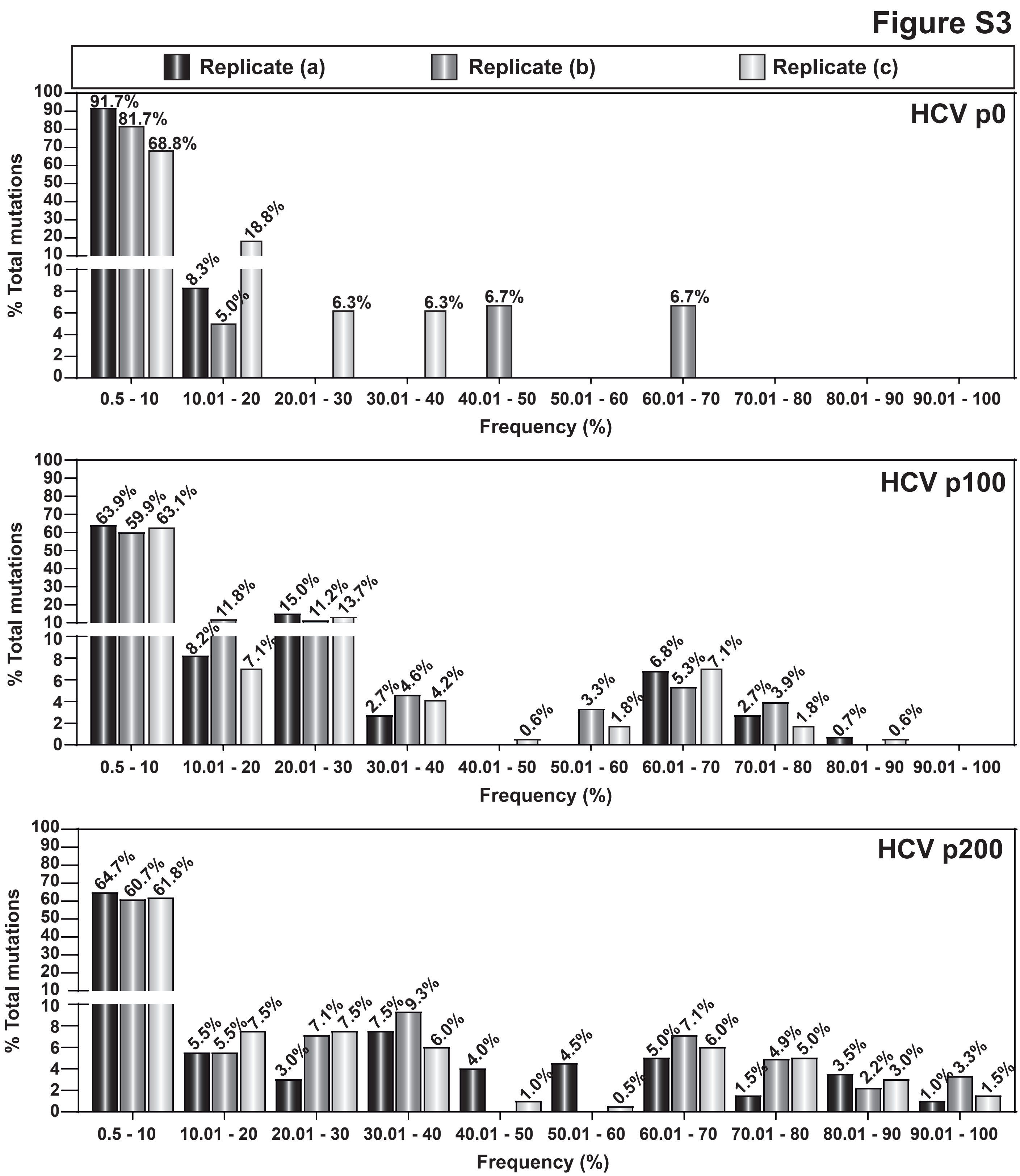

Figure S3. Percentage of total mutations observed in HCV laboratory populations (HCV p0, HCV p100, and HCV p200) distributed according to their frequency with a 0.5% as the low frequency limit. Region analyzed corresponds to genomic residues 7,649 (NS5A-coding region) to 8,653 (NS5B-coding region) (genome numbering according to reference isolate JFH-1, accession number AB047639). Color code of each replicate [(a), (b) and (c)] is indicated on the top box. Data origin and experimental procedures are described in Materials and Methods.
